# Comprehensive serial analysis of gene expression of the cervical transcriptome

**DOI:** 10.1186/1471-2164-8-142

**Published:** 2007-06-01

**Authors:** Ashleen Shadeo, Raj Chari, Greg Vatcher, Jennifer Campbell, Kim M Lonergan, Jasenka Matisic, Dirk van Niekerk, Thomas Ehlen, Dianne Miller, Michele Follen, Wan L Lam, Calum MacAulay

**Affiliations:** 1 Cancer Genetics & Developmental Biology, British Columbia Cancer Research Centre, Vancouver, BC, Canada; 2 Pathology, British Columbia Cancer Agency, Vancouver, BC, Canada; 3 Obstetrics and Gynaecology, The University of British Columbia, Vancouver, BC, Canada; 4 Gynecologic Oncology, British Columbia Cancer Agency, Vancouver, BC, Canada; 5 Gynecologic Oncology, The University of Texas M.D. Anderson Cancer Center, Houston, Texas, USA; 6 Cancer Imaging, British Columbia Cancer Research Centre, Vancouver, BC, Canada

## Abstract

**Background:**

More than half of the approximately 500,000 women diagnosed with cervical cancer worldwide each year will die from this disease. Investigation of genes expressed in precancer lesions compared to those expressed in normal cervical epithelium will yield insight into the early stages of disease. As such, establishing a baseline from which to compare to, is critical in elucidating the abnormal biology of disease. In this study we examine the normal cervical tissue transcriptome and investigate the similarities and differences in relation to CIN III by Long-SAGE (L-SAGE).

**Results:**

We have sequenced 691,390 tags from four L-SAGE libraries increasing the existing gene expression data on cervical tissue by 20 fold. One-hundred and eighteen unique tags were highly expressed in normal cervical tissue and 107 of them mapped to unique genes, most belong to the ribosomal, calcium-binding and keratinizing gene families. We assessed these genes for aberrant expression in CIN III and five genes showed altered expression. In addition, we have identified twelve unique HPV 16 SAGE tags in the CIN III libraries absent in the normal libraries.

**Conclusion:**

Establishing a baseline of gene expression in normal cervical tissue is key for identifying changes in cancer. We demonstrate the utility of this baseline data by identifying genes with aberrant expression in CIN III when compared to normal tissue.

## Background

Approximately 500,000 women are diagnosed with cervical cancer worldwide each year and more than half of them will die from this disease [1]. The highest incidence rates are observed in developing countries where it is the second most prevalent cancer in women and remains a leading cause of cancer related death [1]. Widely implemented screening programs have been responsible for the much lower incidence and mortality rates seen in the developed world. Present day screening methods primarily identify precancer lesions termed cervical intraepithelial neoplasia (CIN). CIN lesions are classified into three subgroups, CIN I, CIN II and CIN III, corresponding to mild, moderate and severe dysplasia/carcinoma *in situ *(CIS), respectively. CIN III lesions have a high likelihood of progression to invasive disease if left untreated [2]. Human Papillomavirus (HPV) has long been established as a necessary but not sufficient cause for cervical carcinoma development. HPV is detected in 99% of invasive disease, 94% of CIN lesions and 46% of normal cervical epithelium [2]. The high risk strains HPV 16 and HPV 18 are most prevalent in invasive disease.

A comprehensive characterization of gene expression of the normal cervical tissue is critical to establish a baseline for comparison against transcriptomes of precancer and cancer. A recent report described the global expression of genes in cervical epithelium using a serial analysis of gene expression (SAGE) based method, enumerating 30,418 sequence tags generated from one normal uterine ectocervical tissue [3]. Another study compared cDNA microarray profiles of cervical tissue to exfoliated cervical cells used in cytology-based cancer screening [4].

In this study, we increased the depth of our understanding of the normal cervical transcriptome and identified gene expression changes in CINIII. We achieved this (i) by using an unbiased Long SAGE (L-SAGE) approach to improve the accuracy of tag-to-gene mapping [5-7], and (ii) by examining 691,390 L-SAGE tags thus increasing publicly available cervical SAGE data by greater than 20 fold.

## Results

**Figure 1:**
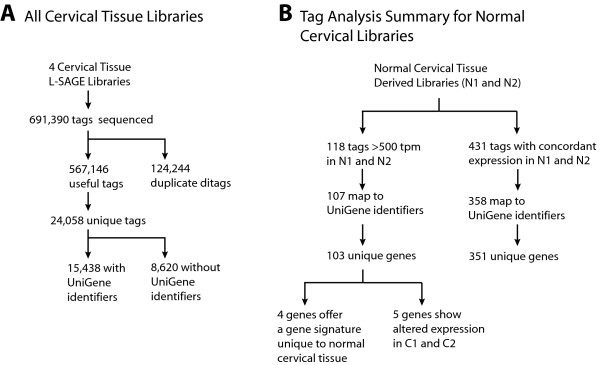
Flow diagram of SAGE analysis and tag-to-gene mapping. A. Sequence tags yielded from the four SAGE libraries were catagorized. Useful tags indicate all sequenced tags less duplicate ditags. B. The abundance and classification of unique tags in the SAGE libraries of normal cervix tissue (N1, N2) are summarized.

In this study, we sequenced 691,390 SAGE tags from four libraries. Cervical L-SAGE libraries N1, N2, C1, and C2 were sequenced to 165,624, 181,224, 173,534, and 171,008 tags, respectively. Duplicate ditags were eliminated from analysis resulting in 136,276, 139,656, 154,828 and 136,386 useful tags respectively and a total of 24, 058 unique tags (Figure 1A). 15,438 of the unique tags mapped to annotated UniGene identifiers. The raw data of the sequence tags have been made publicly available (Gene Expression Omnibus, series accession number GSE6252). We characterized the transcriptome of normal cervical tissue and evaluated the highly expressed genes in terms of tissue specificity, concordant expression among the normal libraries and their altered expression in CIN III lesions (Figure 1B).

### Genes Highly Expressed in Normal Cervical Epithelium

118 unique tags were found to be highly expressed in the normal cervical epithelium (at >500 tpm in both normal libraries). 103 of these tags mapped to UniGene clusters and represent 100 unique genes and hypothetical proteins (Figure 1). Manual examination of tags not mapped by SAGE Genie yielded three additional tags. This results in a total of 107 unique tag-to-gene mappings and 103 unique genes. The abundance of the 118 tags and the genes they represent are summarized in Table 1.

**Table 1:** Tags expressed in normal cervical libraries at ≥500 tags per million.

**Gene Symbol**	**N1 tpm**	**N2 tpm**	**C1 tpm**	**C2 tpm**	**SAGE Tag**
unknown	2510	3623	2655	873	TGATTTCACTTCCACTC
unknown	2517	3430	3307	1672	CTAAGACTTCACCAGTC
unknown	594	559	497	594	GTAGGGGTAAAAGGAGG
unknown	2033	1683	2280	1239	CACCTAATTGGAAGCGC
unknown	807	852	1298	0	CAAGCATCCCCGTTCCA
unknown	822	680	814	631	AGGTGGCAAGAAATGGG
unknown	3757	2714	4134	2757	ACTTTTTCAAAAAAAAA
unknown	616	687	782	374	ACTAACACCCTTAATTC
unknown	1519	1525	2222	2126	AAAAAAAAAAAAAAAAA
unknown	565	780	588	308	TTTAACGGCCGCGGTAC
unknown	6289	6602	6510	4326	TTCATACACCTATCCCC
ACTB	2099	1031	1292	1459	GCTTTATTTGTTTTTTT
ACTG1	1424	1955	1195	1598	CTAGCCTCACGAAACTG
ANXA1	3713	6480	3701	10162	AGAAAGATGTCTATGTA
ANXA2	1644	1754	1298	887	CTTCCAGCTAACAGGTC
AQP3	609	573	930	1136	TTTGCTTTTGTTTTGTT
ATP5G3	543	773	743	851	GGAATGTACGTTATTTC
B2M	8718	2821	9443	5712	GTTGTGGTTAATCTGGT
BZRP	550	960	794	675	GAATTTTATAAGCTGAA
CCNB1IP1	580	924	988	565	CCACTGCACTCCAGCCT
CD24	851	559	775	697	GGAACAAACAGATCGAA
CD74	2348	573	1789	1444	GTTCACATTAGAATAAA
CEACAM7	793	866	891	594	AATCACAAATAAAAGCC
CFL1	1937	1031	1744	1679	GAAGCAGGACCAGTAAG
COX4I1	646	523	969	616	CCTATTTACTGGAAACC
CSTB	712	3881	1227	2676	ATGAGCTGACCTATTTC
CTSD	2502	931	2894	1987	GAAATACAGTTGTTGGC
EEF1A1	2245	1353	1737	1496	TGTGTTGAGAGCTTCTC
EEF1B2	961	1210	1072	1298	GCATTTAAATAAAAGAT
EEF1G	726	501	807	418	TGGGCAAAGCCTTCAAT
FLJ20701	550	501	685	264	ATTTGAGAAGCCTTCGC
FTH1	2311	1275	2325	5176	TTGGGGTTTCCTTTACC
GAPD	1666	2363	2312	2816	TACCATCAATAAAGTAC
GJA1	741	1310	504	513	TGTTCTGGAGAGTGTTC
GLUL	1658	1275	1905	2845	TACAGTATGTTCAAAGT
IGHG1	14118	788	349	4715	GAAATAAAGCACCCACC
IGHG1	18286	773	601	6548	GAAATAAAGCACCCAGC
ITM2B	1203	1031	1369	865	TAAGTAGCAAACAGGGC
K-ALPHA-1	1181	795	1783	1239	TGTACCTGTAATATTTT
KRT13	1380	8206	1815	2046	AAAGCGGGGCTGGAGAA
KRT15	1152	3494	2713	2427	TAATAAAGAATTACTTT
KRT5	2216	1539	1892	1371	GCCCCTGCTGACACGAG
KRT6A	1181	2657	975	1166	AAAGCACAAGTGACTAG
LAMR1	2987	2370	3268	2911	GAAAAATGGTTGATGGA
LGALS7	682	2220	446	425	TAAACCTGCTGTGCGGG
LOC44051	719	702	588	719	GGGTTGGCTTGAAACCA
LY6D	1424	2248	1796	3072	GAGATAAATGATTTAAA
MGC71999	998	1353	924	565	TCACAAGCAAATGTGTC
MYL6	1115	559	995	1063	GTGCTGAATGGCTGAGG
NET-5	1783	780	1298	997	CACAAACGGTAGTTTTG
PERP	1306	2828	1828	2236	CCACAGGAGAATTCGGG
PLCB4	866	752	1072	733	AAAACATTCTCCTCCGC
PPIA	1504	1697	1951	1452	CCTAGCTGGATTGCAGA
PTMA	1049	1017	1518	697	TTCATTATAATCTCAAA
RPL10	2223	1797	1944	1283	AGGGCTTCCAATGTGCT
RPL10A	998	652	1414	821	GGCAAGCCCCAGCGCCT
RPL11	1130	773	1124	829	CGCTGGTTCCAGCAGAA
RPL12	932	580	865	726	ACATCATCGATGACATC
RPL13A	2818	1998	2855	2383	AGGCTACGGAAACAGGC
RPL13A	1490	716	1434	1195	CTCCTCACCTGTATTTT
RPL21	2091	1353	1899	1554	GCATAATAGGTGTTAAA
RPL23	1196	1031	1072	1114	ATTCTCCAGTATATTTG
RPL27A	1923	1310	1989	1899	GAGGGAGTTTCATTAAA
RPL28	1996	1504	1983	1833	GCAGCCATCCGCAGGGC
RPL29	1049	773	1104	814	GGGCTGGGGTCCTCCTG
RPL3	917	702	840	983	GGACCACTGAAGAAAGA
RPL30	1541	1017	1259	961	CCAGAACAGACTGGTGA
RPL31	609	573	568	491	AAGGAGATGGGAACTCC
RPL32	1512	1024	1550	1371	TGCACGTTTTCTGTTTA
RPL35	638	551	665	572	CGCCGCCGGCTCAACAA
RPL36	1570	1275	1628	1173	AGGAAAGCTGCTGCCAA
RPL37	5063	3272	6362	6064	CAATAAATGTTCTGGTT
RPL37A	1350	938	1259	953	AAGACAGTGGCTGGCGG
RPL4	521	537	465	359	CGCCGGAACACCATTCT
RPL41	2649	1812	2609	1906	TTGGTCCTCTGCCCTGG
RPL7	1702	1160	1427	1180	ATTATTTTTCTAAGCTG
RPLP0	815	1239	846	601	CTCAACATCTCCCCCTT
RPLP1	3574	2735	3507	2398	TTCAATAAAAAGCTGAA
RPLP2	2422	1461	2196	2376	GGATTTGGCCTTTTTGA
RPS11	1365	802	917	770	TCTGTACACCTGTCCCC
RPS12	690	695	601	396	GCCGAGGAAGGCATTGC
RPS14	1159	859	1240	843	TAAAAAAAAAAAAAAAA
RPS18	1071	1053	1085	858	TGGTGTTGAGGAAAGCA
RPS19	2598	1604	2357	2618	CTGGGTTAATAAATTGC
RPS20	903	695	1020	667	GCTTTTAAGGATACCGG
RPS23	961	637	833	704	CTGTTGGTGATATTCCT
RPS25	1042	988	924	660	AATAGGTCCAACCAGCT
RPS26	2576	1002	1938	873	TAAGGAGCTGAGTTCTT
RPS27A	815	623	988	887	AACTAAAAAAAAAAAAA
RPS28	998	902	1053	1034	GACGACACGAGCCGATC
RPS29	2245	1940	2325	2317	ATAATTCTTTGTATATA
RPS3A	1218	823	1427	1232	GTGAAGGCAGTAGTTCT
RPS4X	1541	952	1615	924	TCAGATCTTTGTACGTA
RPS8	4021	3587	4282	3996	TAATAAAGGTGTTTATT
RPS9	587	530	407	521	CCAGTGGCCCGGAGCTG
S100A10	514	501	543	506	AGCAGATCAGGACACTT
S100A2	2950	723	2235	3505	GATCTCTTGGGCCCAGG
S100A7	2899	752	388	5990	GAGCAGCGCCCTGTTCC
S100A8	690	1031	827	1466	GCTTTTTTTGTGGGCTG
S100A8	11880	25205	8855	24629	TACCTGCAGAATAATAA
S100A9	13561	17078	10089	18660	GTGGCCACGGCCACAGC
SERPINB3	1137	1454	704	917	CCTTTCTCTCTTTCTCT
SFN	1255	1976	1505	1569	TTTCCTCTCAATAAAGT
SMAD2	2935	2341	3378	1987	CCCATCGTCCTAGAATT
SPINK5	712	4189	0	1305	TCCACCAAGTCTGAGCC
SPRR3	1600	11757	3888	5968	TTTCCTGCTCTGCCCTC
SPRR3	1636	13068	4121	8329	TTTCCTGCTCTTCCCTC
STX3A	646	1905	1318	1312	TAAAATGTTTATGATAA
SUI1	1446	1239	1259	2009	CAATAAACTGAAAAGAG
TAGLN2	660	616	769	543	GTCTGGGGCTTGAGGAA
TMSB4X	2245	616	1175	917	TTGGTGAAGGAAGAAGT
TPI1	947	1382	1589	1584	TGAGGGAATAAACCTGG
TPT1	4726	3659	4915	4568	TAGGTTGTCTAAAAATA
TXNL5	660	838	258	924	TTAGCAATAAATGATGT
UQCRH	646	508	930	726	GGTTTGGCTTAGGCTGG
VAMP8	521	795	459	491	TGGCTGGGAAACTGTTG
VPS13B	763	594	911	0	CACTACTCACCAGACGC
YWHAZ	587	702	517	814	TAAGTGGAATAAAAGTT

**Figure 2:**
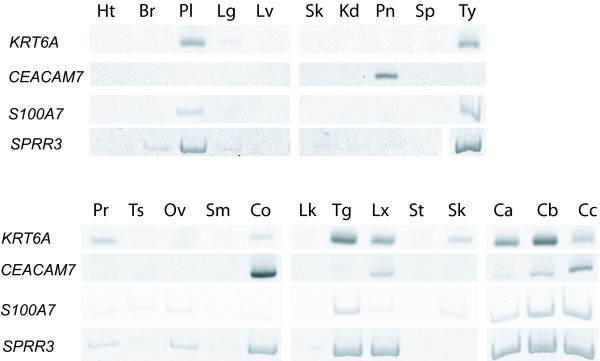
Validation of tissue specificity of gene expression. Reverse transcriptase PCR of four genes in 20 tissue types and three normal cervical specimens. Heart (Ht), breast (Br), placenta (Pl), lung (Lg), liver (Lv), skeletal muscle (Sk), kidney (Kd), pancreas (Pn), spleen (Sp), thymus (Ty), prostate (Pr), testis (Ts), ovary (Ov), small intestine (Sm), colon (Co), peripheral leukocytes (Lk), tongue (Tg) larynx (Lx), stomach (St), skin (Sn). Ca, Cb and Cc are three individual normal cervical tissue specimens.

To determine cervical tissue specific expression, we first investigated the expression of the 107 genes using expression data available at the National Center for Biotechnology Information (NCBI) Unigene database and the National Cancer Institute (NCI) Cancer Genome Anatomy Project (CGAP) SAGE Anatomical Viewer. Based on CGAP information, only four of the 107 genes were unique to cervical tissue: *carcinoembryonic antigen-related cell adhesion molecule 7 *(*CEACAM7*), *keratin 6A *(*KRT6A*), *small proline-rich protein 3 *(*SPRR3*) and *S100 calcium binding protein A7 *(*S100A7*). These genes were further investigated for expression by RT-PCR in 20 different tissue types and three normal cervical specimens (Figure 2). *CEACAM7 *was found to be expressed in colon, larynx, pancreas and two of the three normal cervical specimens. *KRT6A *expression was detected in placenta, thymus, tongue, prostate, larynx, colon, skin and in all three of the normal cervical specimens. *SPRR3 *was found strongly expressed in placenta, thymus, colon, tongue, larynx and all three of the normal cervical cases. *S100A7 *showed expression in placenta, thymus, and tongue and in all three of the normal cervical specimens. All four genes were prominently expressed in the cervical epithelium but this combination of genes was not expressed in the tissues examined (Figure 2).

### Disrupted Gene Expression in CIN III

All tags were assessed for altered expression in CIN III. Four hundred and seventy-six tag show greater than two fold increase in CIN III and are expressed at greater than 15 tpm (see Additional file 1) while 315 tags were decreased in CIN III (see Additional file 2).

**Figure 3:**
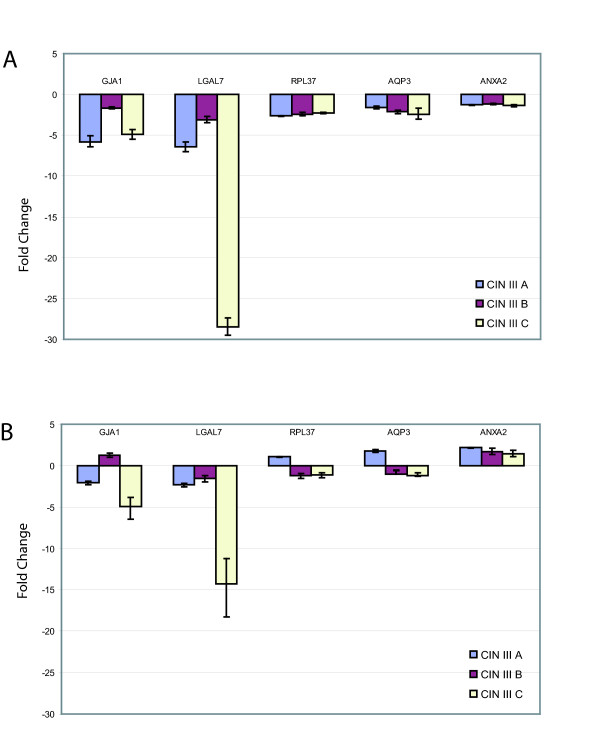
Summary of test panel quantitative PCR results of genes with altered expression in CIN III L-SAGE libraries. A panel of three new CIN III cases (CIN III A, CIN III B, CIN III C) were investigated for expression and compared to three new normal specimens. Gene expression was normalized to *ACTB *and 18S (Figure 3A and 3B, respectively). Zero on the Y-axis denotes mean expression levels of the respective genes in normal cervical tissue. All five genes investigated showed decreased expression.

We determined if the expression of the 107 unique tags, that were highly expressed in normal cervical libraries (> 500 TPM), were disrupted in CIN III. Comparison of expression levels in N1, N2 to the CIN III libraries using the Z-test revealed five differentially expressed genes (Table 2). *Annexin 2 *(*ANXA2*), *galectin 7 *(*LGALS7*) and *connexin 43 *(*GJA1*) exhibited decreased expression in CIN III (Z < -1.96) while *aquaporin 3 *(*AQP3*) and *ribosomal-like protein 37 *(*RPL37*) increased in expression (Z > 1.96). Real-time PCR was performed on a panel of 6 new cervical specimens, three each of normal and CIN III for all five of these genes (Figure 3). Expression results were normalized to housekeeping gene *ACTB and 18S *(Figure 3A and 3B, respectively). Decrease in expression of *ANXA2*, *LGALS7 *and *GJA1 *in CIN III was confirmed while increase in expression of AQP3 and RPL37 were not.

**Table 2:** Highly expressed genes with altered expression in CIN III.

					**Z_Score **^a^				
						
**Tag**	**N1 tpm**	**N2 tpm**	**C1 tpm**	**C2 tpm**	**N1 vs C2**	**N1 vs C1**	**N2 vs C2**	**N2 vs C1**	**Gene Symbol**
TTTGCTTTTGTTTTGTT					-4.7	-3.1	-5.1	-3.5	AQP3
CAATAAATGTTCTGGTT	5063	3272	6362	6064	-3.5	-4.6	-10.8	-12	RPL37
TGTTCTGGAGAGTGTTC	741	1310	504	513	2.4	2.6	6.9	7.3	GJA1
TAAACCTGCTGTGCGGG	682	2220	446	425	2.9	2.7	12.9	13.4	LGALS7
CTTCCAGCTAACAGGTC	1644	1754	1298	887	5.6	2.4	6.3	3.2	ANXA2

^a ^Z > 1.96; reduced expression in CIN III, Z < -1.96; increased expression in CIN III.

### Viral (HPV 16) tags in L-SAGE libraries

HPV transcripts were also detected by L-SAGE. Tags from all four libraries were mapped against the genomes of HPV 16 and HPV 18. While no tags mapped to HPV 18, twelve tags from the CIN III libraries mapped to the more prevalent HPV 16 genome (Table 3). The highest transcript counts of known genes belonged to E5 at 1,180 and 290 tpm and E2 at 240 and 20 tpm, in libraries C2 and C1, respectively. Compared by BLAST [8] against the RefSeq Genome collection, none of the twelve tags matched 100% to the human genome. All twelve tags were also mapped against human transcript sets (mitochondrial genome, RefSeq, UCSC gene set, Unigene, Ensembl, UCSC mRNA, UCSC EST, SAGEmap and SAGEgenie SAGE tag sets). No tags matched to any of the described transcript sets with the exception of CATGCACGCTTTTTAATTACA and CATGTGTATGTATTAAAAATA which mapped to human EST BF909200. The full length EST sequence is 97% identical with the HPV 16 *E5 *gene and was likely amplified from HPV sequences in the originating uterine tumour lesion.

**Table 3:** Tags mapping to HPV16 genome.

Tag	Strand	Gene	N1 tpm	N2 tpm	C1 tpm	C2 tpm
CATGCACGCTTTTTAATTACA	+	E5	0	0	290	1,180
CATGCAACATAAATAAACTTA	+	-	0	0	6	770
CATGGCATTGGACAGGACATA	+	E2	0	0	6	240
CATGTGTATGTATTAAAAATA	-	E5	0	0	6	155
CATGACACAATAGTTACACAA	+	-	0	0	6	125
CATGGGGAGGAATATGATTTA	+	L1/L2	0	0	0	30
CATGTAGACGACACTGCAGTA	-	E2	0	0	0	20
CATGGTAGATTATGGTTTCTG	-	E7	0	0	6	7
CATGTCGTAGGTACTCCTTAA	-	L1/L2	0	0	0	7
CATGGGGATCCTTTGCCCCAG	-	L1/L2	0	0	0	7
CATGATAATATATGTTTGTGC	-	L1/L2	0	0	0	7
CATGCGCCTAGAATGTGCTAT	+	E2	0	0	0	0

## Discussion

This study represents the most comprehensive gene expression analysis of cervical tissue reported to date. In total, 691,390 L-SAGE tags were sequenced (Figure 1A). The length of the L-SAGE tags (21 bp as compared to 14 bp in conventional SAGE) greatly reduces tag-to-gene mapping ambiguity [6]. 107 of the 118 (88%) highly expressed tags (i.e. >500 tpm) were mapped to known genes or hypothetical proteins encompassing 103 unique genes (Figure 1B).

### Assessing Highly Expressed Tags by Functional Group

Of the 107 highly expressed tags (>500 tpm), 47 were expressed at extremely high levels (>10^3 ^tpm) including genes frequently used as controls in expression analysis, *GAPDH *and *ACTB*. High expression of 20 genes in normal cervical tissue was reported in a previous study [3]. Fifteen of these genes are encompassed by our list of 107 high expressers. The most highly expressed tags expressed at >10^4 ^tpm (GTGGCCACGGCCACAGC and TACCTGCAGAATAATAA) mapped to the genes *S100A9 *and *S100A8*, respectively. Both genes belong to the calcium binding protein family. These findings are in agreement with a previous report of high *S100A8 *and *S100A9 *levels in cervical tissue [3]. Although the function of these genes is not well understood, genes within this family have been proposed to participate in a variety of cellular process including cell cycle, wound healing and cell differentiation [9].

**Figure 4:**
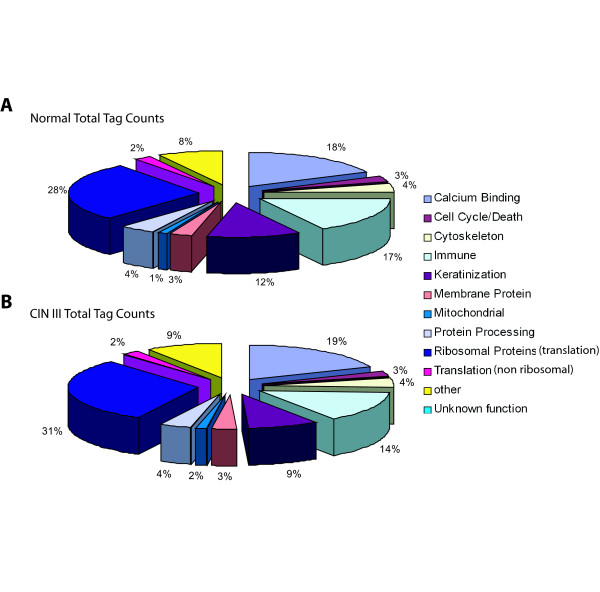
Functional groupings of tags highly expressed (>500 tpm) in normal libraries. Categories are as described. The Other group consists of tags which map to known genes but are not encompassed by any of the ten categories. The Unknown function group consists of tags mapping to no known genes. **A) **Tag counts in the normal libraries are categorized by functional group. **B) **Tags found in A were quantified in both CIN III libraries and categorized according to the same functional grouping scheme. In both groups Ribosomal genes accounted for the greatest number of tags while only the keratins changed in expression or were decreased in the CIN III libraries.

Assigning the 103 highly expressed genes to one of eleven broad functional groups allowed for an assessment of those cellular processes represented by the most abundant transcripts. These cellular processes include calcium binding proteins, cell cycle or cell death, cytoskeleton, immune functioning, keratinization, membrane proteins, mitochondrial, protein processing, translation (ribosomal proteins), translation (non ribosomal proteins) with a small fraction of tags mapping to other functional groups or to genes with no known function (Figure 4A). The 41 ribosomal genes account for the greatest proportion of highly expressed genes at 28% and 31% (normal and CIN III, respectively). In contrast, only five calcium binding genes account for the second largest functional subgroup of highly expressed tags, 18% and 19% (normal and CIN III, respectively).

The relative expression levels of the functional groups do not change greatly between the normal and CIN III libraries however the keratin and immune related functional groups show slight decrease from 12% and 17% in CIN III to 9% and 14% in normal tissue (keratin and immune groups, respectively).

All tags expressed at or greater than 15 tpm in Normal and CIN III libraries (2,814 and 3,279 respectively) were also evaluated according functional group using Onto-Express (see Additional File 3) [10]. The most represented groups included DNA dependent transcription regulators and transcription in both the Normal and CIN III libraries.

### Cervical Tissue Gene Signature

Four of the 103 unique genes we found to be abundantly expressed in normal cervical tissue were documented to have limited or no expression in other tissues according to the web resources NCBI UniGene [11] and NCI CGAP SAGE Anatomical Viewer [12,13] (Figure 1B). These genes (*CEACAM7*, *SPRR3*, *S100A7 *and *KRT6A*) are our candidates for an expression signature unique to normal cervical tissue and were further investigated in a panel of 20 different tissue types and three new normal cervical specimens. We found that all four of these genes were not abundantly expressed simultaneously in any of the 20 tissues examined (Figure 2). Placenta, thymus and tongue were found to express a combination of three genes (*S100A7*, *SPRR3 *and *KRT6A*), while colon expressed another combination (*CEACAM7*, *SPRR3 *and *KRT6A*). *KRT6A *and *SPRR3 *expression was observed in larynx tissue with only minimal expression detected for *S100A7 *and *CEACAM7*. In contrast, two of the cervical cases strongly expressed all four genes investigated while only the third showed very low *CEACAM7 *expression. Significantly, our study is the first to document *CEACAM7 *expression in cervix. The data suggest that abundant expression of *CEACAM7 and S100A7 *collectively, are unique to cervical tissue and have the potential to serve as useful biomarkers in identifying origins of metastatic disease.

It is interesting to note that decreased expression of three of the genes is linked to abnormal growth and organization of epithelium. For example, *CEACAM7 *is a member of the carcinoembryonic antigen family of genes and expression has been documented in highly differentiated normal colon epithelium and the apical surface of normal ductal pancreas epithelium, while loss of expression has been reported in colon hyperplastic polyps [14]. Another member of this gene family, CEACAM1, is shown to have no or very low expression in cervical carcinoma [15]. Decreased expression of *KRT6A *and *S100A7 *have been associated with breast, lung and ovarian cancer [16-20]. *SPRR3 *belongs to the class of small proline rich genes which are expressed in differentiated keratinocytes and has previously been shown to be highly expressed in normal cervical tissue [21].

### Genes Altered in Expression in CIN III

The 107 highly expressed unique tags in the normal libraries were assessed for expression changes in CIN III. Two genes showed increased expression (*AQP3 *and *RPL37*) while three genes declined in transcript counts in the CIN III libraries (*ANXA2*, *GJA1 *and *LGALS7*). All five were evaluated by real-time PCR in a new cervical tissue panel. Results for *ANXA2*, *LGALS7 *and *GJA1 *confirmed L-SAGE findings.

Panel results for *GJA1 *are in agreement with those reported by King *et al. *in that *GJA1 *expression was detected in normal cervical epithelium while reduced expression was observed in CIN III lesions investigated [22]. It has been suggested that this pattern may be a consequence of epithelial disorganization and not causative in dysplasia development [23,24].

The high expression of *LGALS7 *in normal cervical epithelium contrasted by the low expression seen in the CIN III lesions in the tissue panel we report here is similar to those expression patterns seen in studies of other normal tissue types compared to their respective carcinomas including cornea and larynx [25]. *LGALS7 *expression has been hypothesized in all stratified epithelium tissue types and has been experimentally detected in human cornea, heart, larynx, tongue, skin, thymus and thyroid [26-28]. Though, *LGALS7 *has been investigated in the context of cell line models, it is interesting to note that expression of this gene in cervical epithelium has not been previously reported [26-28]. *LGALS7 *is one of fifteen members of the B-galactoside-binding lectin family, some of which have been shown to influence cell growth, cell cycle, apoptosis and cell migration via their predicted role in homeostasis, however, the role of *LGALS7 *in cancer is unclear [27,29]. Support for the pro-apoptotic function of *LGALS7 *was reported by Kuwabara *et al. *and Bernard *et al. *who identified cells more sensitive to apoptosis when *LGALS7 *expression was high in the epithelium derived cells [28,30,31]. In contrast, Demers *et al. *showed an increase in *LGALS7 *expression in lymphoma cells and suggested a positive role in cell growth and dispersal through induced matrix metalloproteinase 9 (*MMP9*) expression [32]. We did not observe a statistically significant change in *MMP9 *in the four libraries investigated. This variance in expression suggests multiple roles for *LGALS7 *that may be tissue-type dependent.

The third gene investigated, *ANXA2*, is known to be highly expressed in epithelial cells and is localized to the plasma membrane and endosome. It has been suggested to function in linking membrane to membrane and membrane to cytoskeleton [33]. The decrease in expression we observe suggests that the loss of *ANXA2 *may be a causative factor in disorganisation of the epithelial architecture, which is characteristic of cervical neoplastic lesions. ANXA2 is also known to bind with S100A10 and participates in transport channel function across the plasma membrane [33]. Interestingly, the ANXA2 binding site on S100A10 also binds NS3, a viral protein from the bluetongue virus, and therefore directly competes with ANXA2. The S100A10 protein has also been shown inhibit Hepatitis B virus polymerase (HBV pol) activity [34]. It is plausible that a S100A10-ANXA2 complex may have a role in HPV infection or viral lifecycle. *S100A10 *expression was high and consistent in Normal and CIN III libraries whereas *ANXA2 *decreased in the CIN III libraries.

The above real-time PCR results were normalized to the widely accepted housekeeping gene *ACTB *(Figure 3A). For comparison we also normalized the genes to a second housekeeping gene 18S (Figure 3B). Briefly, results for *GJA1 *and *LGALS7 *were in agreement to those when normalizing to *ACTB*, however *ANXA2 *was shown to be increased in CIN III as expected by the SAGE data. but this does not concur with the QPCR results when normalized to ACTB. One possible explanation is that on average, the ACTB cycle threshold was >1.3 Ct lower in the CIN III cases indicating an increase in ACTB expression in CIN III lesions. Any Ct decrease less than this in the genes investigated would appear as a decrease in gene expression in CIN III when normalized to ACTB but and increase when normalized to 18S.

The real-time PCR results for *AQP3 *and *RPL37 *did not concur with L-SAGE data and may be due to interindividual differences rather than a representation of changes present in CIN lesions or cancer. It is interesting to note that *RPL37 *overexpression in prostate cancer, colon cancer cell lines and clinical specimens have been reported [35,36]. Our L-SAGE results also suggest a similar pattern in cervical neoplasia. Results for these genes when normalized to 18S showed an increase in expression in only CIN III A (see Additional file 3).

Wong *et al *investigated gene expression in invasive cervical carcinoma by DNA microarray [37]. We investigated this publicly available data through NCBI GEO [38]. Briefly, in this data, GJA1 showed a small decrease in expression in invasive disease while LGALS7 was detected in only four of the 26 specimens, three of which were normal tissue. Moderate AQP3 expression was detected in the majority of cases including the control group. Expression of ANXA2 and RPL37 was not assessed in the microarray study.

### Human L-SAGE Tags Map to the HPV Genome

HPV is an established etiological factor in cervical cancer [2]. There are over 100 known strains of HPV, however HPV 16 and HPV 18 are considered to be the frequent high risk types owing to higher rates of persistent infection, higher rates of progression to cervical neoplasia, and shorter median progression times than other HPV strains [2]. HPV 16 is the most common strain and can be detected in approximately 60% of cervical cancers, while HPV 18 infection occurs in approximately 10–20%. Uncontrolled expression of *E6 *and *E7 *genes from strains 16 or 18 are considered to be essential for oncogenic transformation and function through inhibition of host cell tumour suppressors p53 and the retinoblastoma protein (Rb) [2].

This is the first study to mine human SAGE libraries for viral transcripts. Overall, CIN III library C2 (2,548 tpm) possessed a greater number of tag counts from the more prevalent HPV 16 strain when compared to C1 (320 tpm) (Table 3). HPV 18 tags were not found in any library and no viral tags were detected in the normal libraries. With the exception of two tags, the viral tags expressed in cervical SAGE did not map to known human genes or expressed sequences. The exceptions, CATGCACGCTTTTTAATTACA and CATGTGTATGTATTAAAAATA, mapped to a single human EST isolated from a uterine tumour (EST BF909200). The full length EST sequence is 97% identical with the HPV 16 genome, more specifically the *E5 *gene, and therefore was likely amplified from HPV sequences in the original lesion.

Tags mapping to the *E5 *gene accounted for the greatest proportion of HPV tags mapping to known transcripts in both C1 and C2 (93% and 52%, respectively). *E5 *is considered to be one of three HPV 16 oncogenes (*E5*, *E6 *and *E7*) and is highly expressed in basal cells of premalignant cervical lesions [39]. This expression declines as cells differentiate and move toward the apical face of the epithelium whereas *E6 *and *E7 *expression increases [39]. *E5 *is detected throughout all epithelium layers in high grade lesions such as CIN III. In contrast, expression is restricted to layers closest to the basal cells in low grade lesions, implying that *E5 *expression may be limited to undifferentiated basal cells [40,41]. The high expression of *E5 *we observe in the CIN III libraries and the absence of HPV 16 genes in the normal libraries is in concordance with such studies.

An increase in sample size and inclusion of mild and moderate stages of cervical intraepithelial neoplasia will aid in quantifying the relationship between viral gene expression and disease. This will also assist in further elucidating genes important in early lesion transcriptome events. A comparison of such events with those seen in later stages will help to identify genes important in the molecular pathogenesis of the disease.

## Conclusion

In this study we have described the transcriptome of normal cervical tissues and compared against that of CIN III lesions. This was achieved by construction of four L-SAGE libraries and sequencing to the depth of 172,848 tags per library on average. We highlighted that the Long-SAGE technique provides a comprehensive profile of the transcriptome without focusing on only known genes. Potent tumour suppressors (e.g. *PTEN*), cell cycle mediators (e.g. *CCND1*), and cellular respiration genes (e.g. *NDUFA1*) were found to be tightly regulated in the normal libraries. An expression signature of four highly expressed genes (*KRT6A*, *CEACAM7*, *S100A7 *and *SPRR3*) in normal cervical epithelium was identified and confirmed, and three abundantly expressed genes (*ANXA2*, *GJA1 *and *LGALS7*) were found to have altered expression in CIN III. Furthermore, this is the first study to have identified viral tags in human SAGE libraries demonstrating the versatile nature of SAGE data, which allows for mining and re-mining according to newly posed questions. HPV 16 *E5 *transcripts were found most highly expressed while few *E7 *and no *E6 *transcripts were enumerated.

The identification of expression changes associated with stages of disease progression will help further our understanding of cervical cancer development and potentially elucidate novel targets for diagnosis and treatment. Establishing a baseline from which to compare is essential to the identification of such aberrations and the 20 fold increase in cervical gene expression data presented here is a significant contribution to this effort.

## Methods

### Sample selection

The specimens were collected immediately prior to the LEEP (Loop electrosurgical excision procedure) cone biopsy targeting a small portion of the affected epithelium. These specimens were collected with patient consent at the Vancouver General Hospital Women's Clinic at Vancouver Hospital & Health Science Centre. Cases were assessed by cervical cancer pathologists at Vancouver Hospital and Health Science Centre and were selected without prior knowledge of HPV status. Specimens N1 and N2 in this study were observed to be normal squamous epithelia whereas C1 and C2 were identified as high grade dysplasia or CIN III. Detailed information on specimen pathology based on the LEEP cone specimens can be found in Additional file 4. All samples were stored immediately in RNA *later *and stored at -80°C. Three cases each of CIN III (CIN III A, CIN III B and CIN III C) and normal cervical tissue (NA, NB and NC) which were used for target validation through real-time PCR were also collected, assessed and stored in the same manner.

### L-SAGE Library Construction and Sequence Tag Analysis

The biopsies were individually homogenised in Lysis Binding buffer (100 mM Tris-HCl, pH7.5, 500 mM LiCl, 10 mM EDTA, pH 8.0, 1% LiDS, 5 mM dithiothreitol). Long SAGE libraries were constructed according to the L-SAGE kit manual (Invitrogen, Ontario, Canada). Sequencing was performed at the BC Cancer Agency Michael Smith Genome Sciences Centre. L-SAGE employs 21 basepair sequence tags, reducing the ambiguity in tag-to-gene mapping that is sometimes encountered in classic SAGE libraries which use 14 basepair sequence tags.

### Data Analysis

Tags were mapped using the February 12, 2006 version of *SAGE Genie *[13], and raw tag counts excluding duplicate ditags were normalized to tags per million (tpm). A Z-test analysis, standard for SAGE data analysis, was performed as previously established by Kal *et al. *for comparison of one SAGE library to another using an established cut-off of 1.96 on the absolute Z-score to determine statistically significant differences in expression levels between normal and CIN III [42].

### Reverse Transcriptase PCR

For validation of cervical tissue gene signature, human Multiple Tissue cDNA Panel I and II (Clontech, Mississauga, Ontario) and total RNA for human larynx, skin, stomach and tongue (Stratagene, Cedar Creek, Texas) were used. Five-hundred nanograms from larynx, skin, stomach and tongue was used to generate cDNA. Final concentrations of PCR reagents for cervical tissue signature were 0.5 μM primer, 2 mM MgCl_2_, 0.2 mM dNTP, 1× PCR buffer (Invitrogen), 0.5 U Taq polymerase and 1 μL of cDNA (annealing temperatures: 55°C (*ACTB*, *CEACAM7*), 60°C (*KRT6A*), 65°C (*SPRR3 *and *S100A7*). Total RNA for the panel of six cervical specimens used for validation of genes was isolated using Trizol (Invitrogen) and cDNA was generated using the High Capacity TaqMan Reverse Transcription Reagents, according to the manufacturer's instructions (Applied Biosystems, Foster City, CA).

Expression of genes selected by data analysis were analyzed by real-time PCR using TaqMan^® ^Gene Expression Assays on the ABI 7500 Real-Time PCR System (Applied Biosystems, Foster City, CA), according to manufacturer's instructions. Samples were run in duplicate and normalized against an *beta-actin *(*ACTB*) endogenous control (HuACTB, Applied Biosytems). Assay IDs include *AQP3*, Hs00185020_m1; *GAL7*, Hs00170104_m1; *RPL37*, Hs02340038_g1; and *GJA-1*, Hs00748445_s1. The relative quantification of these target genes in CIN III (CIN III A, CIN III B and CIN III C) samples compared to normal tissue (NA, NB and NC) samples was performed using the established 2^-ΔΔCt ^method, (Applied Biosystems, *Relative Quantitation Of Gene Expression, ABI PRISM 7700 Sequence Detection System: User Bulletin #2*). The cycle threshold (Ct) value of the target gene was normalized to *ACTB *by subtracting the Ct value for *ACTB *from that of the target gene (ΔCt gene = Ct gene - Ct *ACTB*). This number was then averaged from the three normal cases (ave ΔCt normal). The mean fold change of each gene between normal and CIN III was calculated using the following equation: 2 ^-(ΔCt gene - ave ΔCt normal)^. The relative quantification values were then plotted, one indicating no change with respect to normal cervical tissue.

### HPV Tag-to-Gene Mapping

Viral genomic sequence files (viral1.genomic.fna and HPV11.txt) were downloaded from public repositories and processed into 21 bp SAGE tags at every *Nla III *site in both orientations using custom Perl scripts [43,44].

## Abbreviations

Cervical Intraepithelial Neoplasia (CIN), Expressed Sequence Tag (EST), Human Papillomavirus (HPV), Serial Analysis of Gene Expression (SAGE), Tags per million (tpm)

## Authors' contributions

AS designed, organized and performed this study and wrote the manuscript. RC and GV performed data analysis. JC performed gene specific expression assays. KL contributed to the project design, directed library construction and edited the manuscript. JM, DN, TE and DM were responsible for clinical diagnosis, sample acquisition and pathology assessment. MF, WL and CA are principle investigators of this study. All authors read and approved the final draft of this manuscript.

## Supplementary Material

Additional File 1 Supplemental Table 1. Increased Expression in CIN III (>15 tpm and >2 fold change) Click here for file

Additional File 2 Supplemental Table 2. Decreased expression in CIN III (≥15 tpm and ≥2 fold change) Click here for file

Additional File 3 Supplemental Figure 1. Functional annotation of all genes expressed in Normal and CIN III lesions (Supplemental Figure A and B, respectively). Tags expressed >15 TPM included. Click here for file

Additional File 4 Supplemental Table 3. Cervical Specimen Description based on LEEP cone biopsy Pathology Report Click here for file
