# Supplementary material for: Comprehensive serial analysis of gene expression of the cervical transcriptome
Source: BMC Genomics. 2007 Jun 1;8:142. doi: 10.1186/1471-2164-8-142 (PMC1899502; doi:10.1186/1471-2164-8-142)
Supplement: Additional File 1 — Supplemental Table 1. Increased Expression in CIN III (>15 tpm and >2 fold change) [file 1471-2164-8-142-S1.doc]

Supplemental Table 1: Increased Expression in CIN III (>15tpm and >2 fold change)

| **Tag** | **N1** | **N2** | **C1** | **C2** | **Ave N** | **Ave CINIII** | **Fold Change** | **Symbol** |
| --- | --- | --- | --- | --- | --- | --- | --- | --- |
| CACGCTTTTTAATTACA | 0.00 | 0.00 | 290.65 | 1180.47 | 0.00 | 735.56 | 735.56 |  |
| ACACAGCAAGACGAGAA | 0.00 | 0.00 | 658.80 | 513.25 | 0.00 | 586.02 | 586.02 |  |
| CCCGCCTCTTCACGGGC | 0.00 | 0.00 | 180.85 | 117.31 | 0.00 | 149.08 | 149.08 |  |
| GTAATAAGTGTACTATG | 0.00 | 0.00 | 103.34 | 131.98 | 0.00 | 117.66 | 117.66 | SPINK5 |
| TTACATAAGATAAATGT | 0.00 | 0.00 | 148.55 | 29.33 | 0.00 | 88.94 | 88.94 | CXCL10 |
| GGATCCAGGGATCTGGT | 0.00 | 0.00 | 83.96 | 73.32 | 0.00 | 78.64 | 78.64 | KRT24 |
| CTTATGGTCCCCAGCAT | 0.00 | 0.00 | 32.29 | 124.65 | 0.00 | 78.47 | 78.47 | DHRS9 |
| ACAGTCTTGCACATATT | 0.00 | 0.00 | 19.38 | 87.99 | 0.00 | 53.68 | 53.68 | CYP4F3 |
| GACTCTTAAACCTAAAT | 0.00 | 0.00 | 32.29 | 65.99 | 0.00 | 49.14 | 49.14 | CANP |
| ATAGAGGCAATGCATTA | 0.00 | 0.00 | 51.67 | 36.66 | 0.00 | 44.17 | 44.17 | MORF4L2 |
| GGAGAGACAGGTAGTTA | 0.00 | 0.00 | 58.13 | 22.00 | 0.00 | 40.06 | 40.06 | ZFP91 |
| CCCTTCTGTAATAAATA | 0.00 | 0.00 | 25.84 | 43.99 | 0.00 | 34.91 | 34.91 | SERPINB2 |
| GGATTTGGCCTCTTTGA | 0.00 | 0.00 | 25.84 | 43.99 | 0.00 | 34.91 | 34.91 |  |
| CCTTTCCTACCTTCCCT | 0.00 | 0.00 | 38.75 | 29.33 | 0.00 | 34.04 | 34.04 | FLJ36874 |
| TTCGTTTACAGTTCTTA | 0.00 | 0.00 | 38.75 | 29.33 | 0.00 | 34.04 | 34.04 |  |
| AGGGTACGGAAACAGGC | 0.00 | 0.00 | 45.21 | 22.00 | 0.00 | 33.60 | 33.60 |  |
| GATAATGATTTTTTAAC | 0.00 | 0.00 | 45.21 | 22.00 | 0.00 | 33.60 | 33.60 | RAB27A |
| GCACACGTTTACCTATG | 0.00 | 0.00 | 45.21 | 22.00 | 0.00 | 33.60 | 33.60 | LTB4DH |
| CATACACTCTCAAAGCA | 7.34 | 0.00 | 219.60 | 22.00 | 3.67 | 120.80 | 32.92 | CXCL9 |
| CCAACATTCCAGTTTTT | 0.00 | 0.00 | 25.84 | 36.66 | 0.00 | 31.25 | 31.25 | TXN |
| ATACTTTTGGTACAACT | 0.00 | 0.00 | 19.38 | 36.66 | 0.00 | 28.02 | 28.02 | BXDC1 |
| TCAAGTTTATTGCTGCA | 0.00 | 0.00 | 19.38 | 36.66 | 0.00 | 28.02 | 28.02 |  |
| GCTGATCTGTCCAGGGA | 0.00 | 0.00 | 25.84 | 29.33 | 0.00 | 27.58 | 27.58 |  |
| TAAGAAGGTGCAATCCT | 0.00 | 0.00 | 25.84 | 29.33 | 0.00 | 27.58 | 27.58 | MTCBP-1 |
| AGAACAAAGGCAGGCCC | 0.00 | 0.00 | 32.29 | 22.00 | 0.00 | 27.15 | 27.15 | SEC22L1 |
| ATTTTCCAGTATATTTG | 0.00 | 0.00 | 32.29 | 22.00 | 0.00 | 27.15 | 27.15 |  |
| TTAAAACAAACAAAAAT | 0.00 | 0.00 | 32.29 | 22.00 | 0.00 | 27.15 | 27.15 | WAC |
| AGAGTGGTATGTTTCCT | 0.00 | 0.00 | 19.38 | 29.33 | 0.00 | 24.35 | 24.35 |  |
| AGCCAAAAAAAAAAAAC | 0.00 | 0.00 | 19.38 | 29.33 | 0.00 | 24.35 | 24.35 | PCBP2 |
| GAAATAAATGATTTAAA | 0.00 | 0.00 | 19.38 | 29.33 | 0.00 | 24.35 | 24.35 |  |
| TGTATGACTCGTAGTCC | 0.00 | 0.00 | 19.38 | 29.33 | 0.00 | 24.35 | 24.35 | EEF1G |
| AAGAAGTGTGATGAACT | 0.00 | 0.00 | 25.84 | 22.00 | 0.00 | 23.92 | 23.92 |  |
| CACTAATAAAACTGGTT | 0.00 | 0.00 | 25.84 | 22.00 | 0.00 | 23.92 | 23.92 | ZNF518 |
| GGCCTCGGCCTCGGCGG | 0.00 | 0.00 | 25.84 | 22.00 | 0.00 | 23.92 | 23.92 | FRAT2 |
| TAAATTAAAAAAAAAAA | 0.00 | 0.00 | 25.84 | 22.00 | 0.00 | 23.92 | 23.92 |  |
| TTACGAGGAAGAAACTA | 0.00 | 0.00 | 25.84 | 22.00 | 0.00 | 23.92 | 23.92 | SEC13L1 |
| TTGTTGGTCAATGAAAT | 0.00 | 0.00 | 25.84 | 22.00 | 0.00 | 23.92 | 23.92 | KELCHL |
| GCTACTATTAGATCAGG | 7.34 | 0.00 | 103.34 | 65.99 | 3.67 | 84.66 | 23.08 | MCM4 |
| AACACGGTGCTCAGGGG | 0.00 | 0.00 | 19.38 | 22.00 | 0.00 | 20.69 | 20.69 |  |
| ACTGGTCGTCCTAGATT | 0.00 | 0.00 | 19.38 | 22.00 | 0.00 | 20.69 | 20.69 |  |
| AGAATTTTCTAATCTTG | 0.00 | 0.00 | 19.38 | 22.00 | 0.00 | 20.69 | 20.69 | KIAA0528 |
| AGAGAGATTATAAGAAG | 0.00 | 0.00 | 19.38 | 22.00 | 0.00 | 20.69 | 20.69 | FAM33A |
| ATTTTTGAAACCTTATT | 0.00 | 0.00 | 19.38 | 22.00 | 0.00 | 20.69 | 20.69 | DDX3X |
| CAGTCATCTTCAAAAAG | 0.00 | 0.00 | 19.38 | 22.00 | 0.00 | 20.69 | 20.69 |  |
| CGCAGTCTGCTTGTCAA | 0.00 | 0.00 | 19.38 | 22.00 | 0.00 | 20.69 | 20.69 |  |
| GAATAAAAAATTTAGAT | 0.00 | 0.00 | 19.38 | 22.00 | 0.00 | 20.69 | 20.69 | ZNF226 |
| GAGGAATTGGGACCTGG | 0.00 | 0.00 | 19.38 | 22.00 | 0.00 | 20.69 | 20.69 | BACE1 |
| GCAGAAATGATGTACAC | 0.00 | 0.00 | 19.38 | 22.00 | 0.00 | 20.69 | 20.69 | C20orf111 |
| GCTAATGGTGGACGTCT | 0.00 | 0.00 | 19.38 | 22.00 | 0.00 | 20.69 | 20.69 |  |
| TAATAAACTAATATGTC | 0.00 | 0.00 | 19.38 | 22.00 | 0.00 | 20.69 | 20.69 | ASPM |
| TGCTATCATTTTTGTAT | 0.00 | 0.00 | 19.38 | 22.00 | 0.00 | 20.69 | 20.69 | ATP2C1 |
| TGGGGGTTTCCTTTACC | 0.00 | 0.00 | 19.38 | 22.00 | 0.00 | 20.69 | 20.69 | FTH1 |
| TGGTTTTGTATATTAAT | 0.00 | 0.00 | 19.38 | 22.00 | 0.00 | 20.69 | 20.69 | MELK |
| AGGGAAAAAATAAACCC | 0.00 | 21.48 | 77.51 | 337.28 | 10.74 | 207.39 | 19.31 | SPINK5 |
| TGTTTTCAGGTTTCTTT | 7.34 | 0.00 | 96.88 | 22.00 | 3.67 | 59.44 | 16.20 | SIX1 |
| CCTATCAGTAAAAAGAT | 7.34 | 0.00 | 51.67 | 65.99 | 3.67 | 58.83 | 16.03 | MSMB |
| GACCACAAATATTCTTT | 7.34 | 7.16 | 45.21 | 175.97 | 7.25 | 110.59 | 15.26 |  |
| TTATTATTTGTTAAATT | 14.68 | 0.00 | 90.42 | 131.98 | 7.34 | 111.20 | 15.15 |  |
| GGCAGGAGTAGGAATTG | 0.00 | 7.16 | 77.51 | 22.00 | 3.58 | 49.75 | 13.90 | GBP1 |
| CTTATGATCAACATTTA | 7.34 | 0.00 | 32.29 | 65.99 | 3.67 | 49.14 | 13.39 | LSM3 |
| ACATTTTATTAATCCTC | 0.00 | 7.16 | 45.21 | 43.99 | 3.58 | 44.60 | 12.46 | FKSG14 |
| CATTTCTTAAAAAAAAA | 7.34 | 0.00 | 32.29 | 58.66 | 3.67 | 45.48 | 12.39 | PADI1 |
| GGGCTCACCTGGTGGAT | 0.00 | 7.16 | 51.67 | 36.66 | 3.58 | 44.17 | 12.34 | CDT1 |
| TAATAAAAAATTACTTT | 0.00 | 7.16 | 51.67 | 36.66 | 3.58 | 44.17 | 12.34 |  |
| TTGCTCACACAAAAAAA | 0.00 | 7.16 | 64.59 | 22.00 | 3.58 | 43.29 | 12.09 | MYR8 |
| CTTCTGTTTTTGTAAAA | 7.34 | 0.00 | 64.59 | 22.00 | 3.67 | 43.29 | 11.80 |  |
| TGATTAAAACAAGTTGC | 7.34 | 0.00 | 32.29 | 51.32 | 3.67 | 41.81 | 11.40 | INSIG1 |
| GACTGTTGCTGCTCCCT | 0.00 | 7.16 | 58.13 | 22.00 | 3.58 | 40.06 | 11.19 | CDW92 |
| TACTTGGTCTTTTGTGT | 7.34 | 0.00 | 51.67 | 29.33 | 3.67 | 40.50 | 11.04 | TMPO |
| CATAACAATAATGCTAG | 7.34 | 0.00 | 25.84 | 51.32 | 3.67 | 38.58 | 10.52 | CMAH |
| GTCATTTGGATCATCTT | 0.00 | 7.16 | 45.21 | 29.33 | 3.58 | 37.27 | 10.41 | C20orf24 |
| TCGTAATAGTTGATGTA | 7.34 | 0.00 | 32.29 | 43.99 | 3.67 | 38.14 | 10.40 | MIF |
| CTCACAAGTTTTGGGAA | 7.34 | 0.00 | 45.21 | 29.33 | 3.67 | 37.27 | 10.16 | NUP160 |
| TCTGCATCTTGGACGCC | 14.68 | 0.00 | 77.51 | 65.99 | 7.34 | 71.75 | 9.78 | FTHFD |
| TCCAATAAAAAGCTGAA | 0.00 | 7.16 | 32.29 | 36.66 | 3.58 | 34.48 | 9.63 |  |
| AGCAAATAAATGGCTGT | 0.00 | 7.16 | 38.75 | 29.33 | 3.58 | 34.04 | 9.51 |  |
| GAATGTTTTTTCCTGAT | 0.00 | 7.16 | 38.75 | 29.33 | 3.58 | 34.04 | 9.51 | PLXNA3 |
| AGGAACAACTGACCTGT | 7.34 | 14.32 | 58.13 | 146.64 | 10.83 | 102.39 | 9.45 | KLK12 |
| AGAGAAGAATGAAGAGA | 7.34 | 0.00 | 32.29 | 36.66 | 3.67 | 34.48 | 9.40 | NMU |
| GCTGAAATAAAACTAAA | 0.00 | 7.16 | 45.21 | 22.00 | 3.58 | 33.60 | 9.39 | NCBP1 |
| GTTTCTCAAATTTAGTT | 0.00 | 7.16 | 45.21 | 22.00 | 3.58 | 33.60 | 9.39 | RNF6 |
| CCCGCATAGATGCCGCG | 22.01 | 0.00 | 109.80 | 95.32 | 11.01 | 102.56 | 9.32 | CDKN2A |
| ATCACCAAGTGAAAACA | 7.34 | 0.00 | 38.75 | 29.33 | 3.67 | 34.04 | 9.28 | K-ALPHA-1 |
| TCCGAGACTGCTCCTGC | 14.68 | 0.00 | 58.13 | 73.32 | 7.34 | 65.73 | 8.96 | HIST1H1C |
| TACATTTTATGTAAATA | 0.00 | 7.16 | 19.38 | 43.99 | 3.58 | 31.68 | 8.85 | PVRL4 |
| ATAAAATTCCACAAAAT | 0.00 | 7.16 | 25.84 | 36.66 | 3.58 | 31.25 | 8.73 | SORT1 |
| AATTTAGAGCATTCCAC | 0.00 | 7.16 | 32.29 | 29.33 | 3.58 | 30.81 | 8.61 | ARL10C |
| GTTGTAGACTTTCACCT | 0.00 | 7.16 | 32.29 | 29.33 | 3.58 | 30.81 | 8.61 | MGC24665 |
| TGTCACACACAGACCCA | 7.34 | 0.00 | 25.84 | 36.66 | 3.67 | 31.25 | 8.52 | PGR1 |
| AAGGTAACTAACGGAAG | 0.00 | 7.16 | 38.75 | 22.00 | 3.58 | 30.37 | 8.48 | DHFR |
| ATTTACCTGCTAATAAA | 0.00 | 7.16 | 38.75 | 22.00 | 3.58 | 30.37 | 8.48 |  |
| GTGGCCCCGGCCGCACC | 0.00 | 7.16 | 38.75 | 22.00 | 3.58 | 30.37 | 8.48 | ALG12 |
| CTCCACCCGAGGAAAAA | 14.68 | 0.00 | 58.13 | 65.99 | 7.34 | 62.06 | 8.46 |  |
| TGAAATACTGAAAAAAA | 14.68 | 0.00 | 71.05 | 51.32 | 7.34 | 61.19 | 8.34 | PLEKHA2 |
| GCATTGTGACTAATGGC | 7.34 | 0.00 | 38.75 | 22.00 | 3.67 | 30.37 | 8.28 | DONSON |
| TGCACCTTGGGGTTTTG | 7.34 | 0.00 | 38.75 | 22.00 | 3.67 | 30.37 | 8.28 | CDK2 |
| GAGTAGTAATTTAAAGA | 0.00 | 14.32 | 83.96 | 29.33 | 7.16 | 56.65 | 7.91 | BCCIP |
| TTCACTTACATTTGGCT | 7.34 | 7.16 | 77.51 | 36.66 | 7.25 | 57.08 | 7.87 | APOL6 |
| CTCCACCCGAGAAAAAA | 22.01 | 0.00 | 77.51 | 95.32 | 11.01 | 86.41 | 7.85 |  |
| CCTGATCTGCGGTGTCC | 14.68 | 0.00 | 71.05 | 43.99 | 7.34 | 57.52 | 7.84 | MUC16 |
| GTTAATAAATGTGGTTT | 14.68 | 0.00 | 71.05 | 43.99 | 7.34 | 57.52 | 7.84 | KIF11 |
| GAAAAAATGGTTGATGG | 0.00 | 7.16 | 19.38 | 36.66 | 3.58 | 28.02 | 7.83 |  |
| AGATTATATTGGTTTCC | 0.00 | 7.16 | 25.84 | 29.33 | 3.58 | 27.58 | 7.70 | UGDH |
| GGAGGCAGGTGGGGCTT | 0.00 | 7.16 | 25.84 | 29.33 | 3.58 | 27.58 | 7.70 | B4GALT2 |
| TGTAAGTTACTAATATA | 0.00 | 7.16 | 25.84 | 29.33 | 3.58 | 27.58 | 7.70 | CPNE3 |
| TTCCCCGTGACAGGTGG | 0.00 | 7.16 | 25.84 | 29.33 | 3.58 | 27.58 | 7.70 | PPP1CA |
| TTTAAAATAGAAGTGAC | 0.00 | 7.16 | 25.84 | 29.33 | 3.58 | 27.58 | 7.70 | OSBPL2 |
| ACCAAAATATCACAGGC | 7.34 | 0.00 | 19.38 | 36.66 | 3.67 | 28.02 | 7.64 | MGC4677 |
| CGCCCCCACAGCGGCCC | 7.34 | 0.00 | 19.38 | 36.66 | 3.67 | 28.02 | 7.64 | LOC112869 |
| CCTATTTTCTTTGAGGT | 0.00 | 7.16 | 32.29 | 22.00 | 3.58 | 27.15 | 7.58 | SMC5L1 |
| TCTAAAATTAAATTTCA | 7.34 | 7.16 | 71.05 | 29.33 | 7.25 | 50.19 | 6.92 |  |
| CTGCAGGGCCAAAAGGA | 0.00 | 7.16 | 19.38 | 29.33 | 3.58 | 24.35 | 6.80 |  |
| TATGCCTTTTTGTTAGA | 0.00 | 7.16 | 19.38 | 29.33 | 3.58 | 24.35 | 6.80 |  |
| AAAACATTCTCCTCAAA | 0.00 | 7.16 | 25.84 | 22.00 | 3.58 | 23.92 | 6.68 |  |
| CCCATCGTCCTAGGATT | 0.00 | 7.16 | 25.84 | 22.00 | 3.58 | 23.92 | 6.68 |  |
| TAGCTGCCTTTGTTACT | 0.00 | 7.16 | 25.84 | 22.00 | 3.58 | 23.92 | 6.68 | DVL2 |
| GGGAGCCTCACAGCAGC | 0.00 | 14.32 | 51.67 | 43.99 | 7.16 | 47.83 | 6.68 | TPX2 |
| AAGCCTTAGCTGAAATG | 7.34 | 0.00 | 19.38 | 29.33 | 3.67 | 24.35 | 6.64 |  |
| ATGTGAAACTGGGGTGC | 7.34 | 0.00 | 19.38 | 29.33 | 3.67 | 24.35 | 6.64 | REPS2 |
| CGGATGATTGATGGGAA | 7.34 | 0.00 | 19.38 | 29.33 | 3.67 | 24.35 | 6.64 | SLC25A11 |
| GAGAGTGGGGCCAAAGG | 7.34 | 0.00 | 19.38 | 29.33 | 3.67 | 24.35 | 6.64 | RPS3 |
| CTTGATTAAACCATTAA | 14.68 | 0.00 | 38.75 | 58.66 | 7.34 | 48.70 | 6.64 | C6orf83 |
| AAGTACTTCATTTTGAT | 0.00 | 57.28 | 193.76 | 183.30 | 28.64 | 188.53 | 6.58 |  |
| CACACCCCTGAACCACG | 7.34 | 0.00 | 25.84 | 22.00 | 3.67 | 23.92 | 6.52 | PGRMC1 |
| CGTGAACAATTTAAAAA | 7.34 | 0.00 | 25.84 | 22.00 | 3.67 | 23.92 | 6.52 | C2orf33 |
| TGTCTTTGTGTTTATGG | 7.34 | 0.00 | 25.84 | 22.00 | 3.67 | 23.92 | 6.52 |  |
| TTTATAAAATAAATCTT | 7.34 | 0.00 | 25.84 | 22.00 | 3.67 | 23.92 | 6.52 |  |
| TTTTATCTTTTTTTGTT | 7.34 | 0.00 | 25.84 | 22.00 | 3.67 | 23.92 | 6.52 | KLHL13 |
| GGACCTTTATTCGTTAA | 44.03 | 0.00 | 135.63 | 146.64 | 22.01 | 141.14 | 6.41 |  |
| TGTTCCATTTATTTTGT | 22.01 | 0.00 | 45.21 | 95.32 | 11.01 | 70.26 | 6.38 | HLA-B |
| GTAAGTGTACTGGAGTG | 36.69 | 0.00 | 122.72 | 109.98 | 18.35 | 116.35 | 6.34 |  |
| CTGTATGTTTAGATTTG | 14.68 | 0.00 | 38.75 | 51.32 | 7.34 | 45.04 | 6.14 | LOC132321 |
| TAAGTTCCTTCCCCCTC | 7.34 | 7.16 | 58.13 | 29.33 | 7.25 | 43.73 | 6.03 | XTP3TPA |
| CTCGCGCTGGGCAGGGA | 14.68 | 0.00 | 51.67 | 36.66 | 7.34 | 44.17 | 6.02 | CLDN3 |
| CTCCACCCGAGGACAAA | 29.35 | 0.00 | 109.80 | 65.99 | 14.68 | 87.89 | 5.99 |  |
| AGAATCGCTTGAGCCCA | 7.34 | 7.16 | 64.59 | 22.00 | 7.25 | 43.29 | 5.97 | MRPS11 |
| TTTTTTGAAAGCACTGG | 7.34 | 7.16 | 64.59 | 22.00 | 7.25 | 43.29 | 5.97 | PLSCR1 |
| AAATTAAAACAGACCTA | 0.00 | 14.32 | 32.29 | 51.32 | 7.16 | 41.81 | 5.84 | RNF103 |
| AAATAATGTTGGCATTA | 0.00 | 7.16 | 19.38 | 22.00 | 3.58 | 20.69 | 5.78 | SREBF2 |
| AAGTGGATAGATACTTC | 0.00 | 7.16 | 19.38 | 22.00 | 3.58 | 20.69 | 5.78 |  |
| ATGGGTCAGAAACAAAT | 0.00 | 7.16 | 19.38 | 22.00 | 3.58 | 20.69 | 5.78 | MAPK1 |
| GAAACCCTGAATTCTCC | 0.00 | 7.16 | 19.38 | 22.00 | 3.58 | 20.69 | 5.78 |  |
| TAGGTTGTATAAAAATA | 0.00 | 7.16 | 19.38 | 22.00 | 3.58 | 20.69 | 5.78 |  |
| TATTTTTTTACAGTCTA | 0.00 | 7.16 | 19.38 | 22.00 | 3.58 | 20.69 | 5.78 | SFRS10 |
| TGCCATCTGTACATATT | 0.00 | 7.16 | 19.38 | 22.00 | 3.58 | 20.69 | 5.78 | CCNB1 |
| TGTTCACACTGTGTGTA | 0.00 | 7.16 | 19.38 | 22.00 | 3.58 | 20.69 | 5.78 | MGC15396 |
| TTGTTTAACAACTTTTC | 7.34 | 7.16 | 38.75 | 43.99 | 7.25 | 41.37 | 5.71 |  |
| GAAGCTACACCCCTACA | 14.68 | 0.00 | 32.29 | 51.32 | 7.34 | 41.81 | 5.70 | CCDC3 |
| TTTTCTGAAAATATAAC | 51.37 | 85.93 | 329.40 | 447.26 | 68.65 | 388.33 | 5.66 | TXN |
| GGCATTTTAAAAAAAAA | 0.00 | 14.32 | 51.67 | 29.33 | 7.16 | 40.50 | 5.66 | TFDP1 |
| TAAATAAATGTTTTCCT | 7.34 | 7.16 | 45.21 | 36.66 | 7.25 | 40.94 | 5.65 | RAD1 |
| AGCCACCACGCCCGGCC | 7.34 | 0.00 | 19.38 | 22.00 | 3.67 | 20.69 | 5.64 | PPFIA1 |
| CCTAACACAGCATTCCC | 7.34 | 0.00 | 19.38 | 22.00 | 3.67 | 20.69 | 5.64 | FLJ23311 |
| CCTGTCTTGGCTGCAGT | 7.34 | 0.00 | 19.38 | 22.00 | 3.67 | 20.69 | 5.64 | TUSC1 |
| CTGAAATTCGGTGCAGC | 7.34 | 0.00 | 19.38 | 22.00 | 3.67 | 20.69 | 5.64 | CSNK1E |
| CTTAGTTTGTTCCAGGG | 7.34 | 0.00 | 19.38 | 22.00 | 3.67 | 20.69 | 5.64 |  |
| CTTTAAGGTTTATGTAA | 7.34 | 0.00 | 19.38 | 22.00 | 3.67 | 20.69 | 5.64 | TBC1D15 |
| GAATAAACATTCTCAGC | 7.34 | 0.00 | 19.38 | 22.00 | 3.67 | 20.69 | 5.64 | PDLIM7 |
| GCCAGCAAAAAAAAAAA | 7.34 | 0.00 | 19.38 | 22.00 | 3.67 | 20.69 | 5.64 |  |
| GCCTCTTTCCTTGGACA | 7.34 | 0.00 | 19.38 | 22.00 | 3.67 | 20.69 | 5.64 | SLU7 |
| GGAACCCTGACCAGGTC | 7.34 | 0.00 | 19.38 | 22.00 | 3.67 | 20.69 | 5.64 | ASH2L |
| GTCACAGTCCTATGTAC | 7.34 | 0.00 | 19.38 | 22.00 | 3.67 | 20.69 | 5.64 | SRF |
| TCCAGTATGAGTGGTGG | 7.34 | 0.00 | 19.38 | 22.00 | 3.67 | 20.69 | 5.64 | HNRPH1 |
| TGTACAAAATCTACTTC | 7.34 | 0.00 | 19.38 | 22.00 | 3.67 | 20.69 | 5.64 | DKFZp547B1713 |
| ACCGAAACTTGATATTT | 14.68 | 0.00 | 38.75 | 43.99 | 7.34 | 41.37 | 5.64 | PDXK |
| TAATTGCCATCTGTCAC | 7.34 | 7.16 | 51.67 | 29.33 | 7.25 | 40.50 | 5.59 | NUSAP1 |
| GAGAGGGCAGACTGTGC | 14.68 | 0.00 | 45.21 | 36.66 | 7.34 | 40.94 | 5.58 | RNF26 |
| ATGTAGAGTGTGGTTAT | 7.34 | 7.16 | 58.13 | 22.00 | 7.25 | 40.06 | 5.53 | TYMS |
| CACCTAGCATAGTGCTT | 14.68 | 7.16 | 51.67 | 65.99 | 10.92 | 58.83 | 5.39 | DHX40 |
| CTTCAAGGCCGGGGCAG | 7.34 | 7.16 | 32.29 | 43.99 | 7.25 | 38.14 | 5.26 | DGCR8 |
| TTCCAGCCAATGGATGA | 7.34 | 7.16 | 32.29 | 43.99 | 7.25 | 38.14 | 5.26 | MGC5576 |
| CTTCCTTGTAGTCCAGC | 7.34 | 7.16 | 38.75 | 36.66 | 7.25 | 37.71 | 5.20 | KIAA1434 |
| GTCTTCTTAATTTTCTA | 7.34 | 7.16 | 38.75 | 36.66 | 7.25 | 37.71 | 5.20 | DNAJD1 |
| GACTCGCCCACGCCTCG | 14.68 | 0.00 | 32.29 | 43.99 | 7.34 | 38.14 | 5.20 | MCM5 |
| GTTAAATGACTTCCCTT | 29.35 | 0.00 | 71.05 | 80.65 | 14.68 | 75.85 | 5.17 | LOC440712 |
| TGTCCTGGTTCCCGTTT | 36.69 | 7.16 | 116.26 | 109.98 | 21.93 | 113.12 | 5.16 | CDKN1A |
| TGCGGCTGGTTTTGGAC | 7.34 | 7.16 | 51.67 | 22.00 | 7.25 | 36.83 | 5.08 | DCTN1 |
| CTGGGACTGACAGCCTG | 36.69 | 0.00 | 90.42 | 95.32 | 18.35 | 92.87 | 5.06 | LSM4 |
| GGCCCTCTGAGCAACTG | 7.34 | 7.16 | 19.38 | 51.32 | 7.25 | 35.35 | 4.88 | PIN1 |
| GCCTCTGCCAGATGGCT | 0.00 | 14.32 | 32.29 | 36.66 | 7.16 | 34.48 | 4.81 | BAP1 |
| TTGAAAGGTTTCTATGG | 29.35 | 7.16 | 64.59 | 109.98 | 18.26 | 87.28 | 4.78 | UAP1 |
| TGCAAAGTACTGTTAAG | 7.34 | 7.16 | 32.29 | 36.66 | 7.25 | 34.48 | 4.76 |  |
| TGCCATCTGTACATAAA | 7.34 | 7.16 | 32.29 | 36.66 | 7.25 | 34.48 | 4.76 |  |
| CAATAAATGCTCTGGTT | 7.34 | 14.32 | 51.67 | 51.32 | 10.83 | 51.50 | 4.76 |  |
| CTCCCTCCTCTCCTACC | 0.00 | 14.32 | 38.75 | 29.33 | 7.16 | 34.04 | 4.75 | TK1 |
| TAAGCATTAAACAGTGT | 14.68 | 0.00 | 32.29 | 36.66 | 7.34 | 34.48 | 4.70 | SDCBP |
| TACTGTAGACATCAAAC | 14.68 | 0.00 | 32.29 | 36.66 | 7.34 | 34.48 | 4.70 | MIR16 |
| GACCCTTTTGGCCCTGC | 7.34 | 7.16 | 38.75 | 29.33 | 7.25 | 34.04 | 4.70 | FLJ21019 |
| GATGTTGTCCATATTAA | 7.34 | 7.16 | 38.75 | 29.33 | 7.25 | 34.04 | 4.70 | ROD1 |
| TAATATATAATTTAAAA | 14.68 | 14.32 | 77.51 | 58.66 | 14.50 | 68.08 | 4.70 | FCHO2 |
| ATTTAGTCATAATTGTG | 14.68 | 21.48 | 103.34 | 65.99 | 18.08 | 84.66 | 4.68 | IFI44 |
| ATGTTTTTAAAATCTTA | 7.34 | 7.16 | 45.21 | 22.00 | 7.25 | 33.60 | 4.64 | FMR1 |
| CAGTAGACAGAAGAGGA | 7.34 | 7.16 | 45.21 | 22.00 | 7.25 | 33.60 | 4.64 | PFDN1 |
| CTTTTATGTTTTTCAGA | 7.34 | 7.16 | 45.21 | 22.00 | 7.25 | 33.60 | 4.64 | AGPAT5 |
| TGTGTGCGCTCGCAGAC | 7.34 | 7.16 | 45.21 | 22.00 | 7.25 | 33.60 | 4.64 | STARD7 |
| TGTTCTATGAATAAAGA | 7.34 | 14.32 | 71.05 | 29.33 | 10.83 | 50.19 | 4.63 | KIAA0186 |
| TGAGCTTAATGCAGAAT | 14.68 | 7.16 | 32.29 | 65.99 | 10.92 | 49.14 | 4.50 |  |
| CAAGACAGAAACAAAAG | 0.00 | 21.48 | 51.67 | 43.99 | 10.74 | 47.83 | 4.45 | HSPC150 |
| CTCTTAATGTATTCCTT | 0.00 | 21.48 | 51.67 | 43.99 | 10.74 | 47.83 | 4.45 | YWHAQ |
| TGGACAGTGCACGTGCC | 14.68 | 21.48 | 116.26 | 43.99 | 18.08 | 80.13 | 4.43 | EYA2 |
| CTTGTAATCCTACTTGG | 80.72 | 14.32 | 219.60 | 197.97 | 47.52 | 208.78 | 4.39 | G1P3 |
| GTTTGATTTTGACCTGG | 7.34 | 21.48 | 45.21 | 80.65 | 14.41 | 62.93 | 4.37 | HK1 |
| ATGTTGATTTTTTTTCA | 14.68 | 7.16 | 58.13 | 36.66 | 10.92 | 47.39 | 4.34 | PSMD5 |
| TGTTCTGATTTTTGGTT | 7.34 | 21.48 | 51.67 | 73.32 | 14.41 | 62.50 | 4.34 | ACOX1 |
| CCTGTAATCCCAGCCAG | 7.34 | 14.32 | 64.59 | 29.33 | 10.83 | 46.96 | 4.34 | ERBB3 |
| AGTTTGAAATACTGCTC | 7.34 | 7.16 | 25.84 | 36.66 | 7.25 | 31.25 | 4.31 |  |
| TCAACAGCGTTCCTAGA | 7.34 | 7.16 | 25.84 | 36.66 | 7.25 | 31.25 | 4.31 | BID |
| TGCAGACCCATTTTGAT | 0.00 | 14.32 | 32.29 | 29.33 | 7.16 | 30.81 | 4.30 | TAX1BP1 |
| GTTCTCTTTGTACGATA | 7.34 | 7.16 | 32.29 | 29.33 | 7.25 | 30.81 | 4.25 |  |
| TGCCCTCAAAAAAAAAG | 7.34 | 7.16 | 32.29 | 29.33 | 7.25 | 30.81 | 4.25 |  |
| TGACCTGTTACTTCAGG | 7.34 | 14.32 | 32.29 | 58.66 | 10.83 | 45.48 | 4.20 | LOC196264 |
| ACAAGCATTTTGTGAAC | 7.34 | 7.16 | 38.75 | 22.00 | 7.25 | 30.37 | 4.19 | CDKN2A |
| TAGAGTGTAAAAAAAAA | 7.34 | 7.16 | 38.75 | 22.00 | 7.25 | 30.37 | 4.19 | PDPK1 |
| CTCCACCCGAAAAAAAA | 330.21 | 7.16 | 723.38 | 689.22 | 168.69 | 706.30 | 4.19 |  |
| AGGTCTGCCAGAAGGCC | 14.68 | 28.64 | 71.05 | 109.98 | 21.66 | 90.51 | 4.18 | AKR1C2 |
| CTGGCGAGCGCGATAAG | 7.34 | 35.80 | 83.96 | 95.32 | 21.57 | 89.64 | 4.16 | UBE2S |
| TAATTCTTAAACTGGGC | 0.00 | 21.48 | 45.21 | 43.99 | 10.74 | 44.60 | 4.15 | BAG5 |
| GCCTGTACAACCTCAAA | 14.68 | 7.16 | 38.75 | 51.32 | 10.92 | 45.04 | 4.13 | IGFBP2 |
| GTCTTAACTCACCGAAA | 14.68 | 7.16 | 45.21 | 43.99 | 10.92 | 44.60 | 4.09 | TXNL4A |
| AAAAAATGAAAAGTAGA | 7.34 | 14.32 | 51.67 | 36.66 | 10.83 | 44.17 | 4.08 | MYO5C |
| TGGACCAGGCGCCCAGC | 7.34 | 14.32 | 51.67 | 36.66 | 10.83 | 44.17 | 4.08 | GPR108 |
| TAATTGCAGATAACTTT | 7.34 | 14.32 | 58.13 | 29.33 | 10.83 | 43.73 | 4.04 | CDKN2C |
| GAAATCAAAAAAAAAAA | 22.01 | 7.16 | 51.67 | 65.99 | 14.59 | 58.83 | 4.03 | TPSB2 |
| AGCTGTATTCTTCACAG | 22.01 | 14.32 | 83.96 | 58.66 | 18.17 | 71.31 | 3.93 | CKS2 |
| TGTTTGTACATTTTTGT | 44.03 | 28.64 | 142.09 | 139.31 | 36.34 | 140.70 | 3.87 | B4GALT5 |
| TAGCTGAGACATAAATT | 22.01 | 14.32 | 45.21 | 95.32 | 18.17 | 70.26 | 3.87 | KPNA2 |
| GCAAGCCATAGCTAGGC | 7.34 | 7.16 | 19.38 | 36.66 | 7.25 | 28.02 | 3.87 | S100A11 |
| ACTTTTTCAAAAAAACA | 7.34 | 14.32 | 38.75 | 43.99 | 10.83 | 41.37 | 3.82 |  |
| GGAGGGAGTTTCATTAA | 7.34 | 14.32 | 38.75 | 43.99 | 10.83 | 41.37 | 3.82 |  |
| CTCCTCACCTGTCTAGG | 7.34 | 21.48 | 58.13 | 51.32 | 14.41 | 54.73 | 3.80 | BAK1 |
| GGCAACAAGAGCGAAAC | 7.34 | 21.48 | 58.13 | 51.32 | 14.41 | 54.73 | 3.80 |  |
| CGTGAGCCACTGCGCCC | 22.01 | 21.48 | 83.96 | 80.65 | 21.75 | 82.31 | 3.78 | TRIM16 |
| AAAAAAAAGAAAAAAAA | 7.34 | 14.32 | 45.21 | 36.66 | 10.83 | 40.94 | 3.78 | RPL36AL |
| GGTGGATGTGCTTGGAA | 22.01 | 7.16 | 58.13 | 51.32 | 14.59 | 54.73 | 3.75 | MBD3 |
| TACCATCAATAAGGTAC | 7.34 | 7.16 | 32.29 | 22.00 | 7.25 | 27.15 | 3.74 |  |
| TATTGGTTGGTGGTCCT | 7.34 | 7.16 | 32.29 | 22.00 | 7.25 | 27.15 | 3.74 | CALM1 |
| GGCCCTGGTGTTTGCAC | 14.68 | 14.32 | 38.75 | 65.99 | 14.50 | 52.37 | 3.61 | GAK |
| CCTAATGTGTTCTTTTT | 29.35 | 7.16 | 64.59 | 65.99 | 18.26 | 65.29 | 3.58 | LMNB2 |
| GCAGGCGGCTCTGGCTT | 7.34 | 21.48 | 51.67 | 51.32 | 14.41 | 51.50 | 3.57 |  |
| AGTGTCCGGCATCTGCT | 22.01 | 14.32 | 71.05 | 58.66 | 18.17 | 64.85 | 3.57 | PDXK |
| GCACCTTATTGTTTTGA | 14.68 | 14.32 | 51.67 | 51.32 | 14.50 | 51.50 | 3.55 | FLJ14668 |
| GAGGAAGAAGAAGCAGC | 22.01 | 7.16 | 51.67 | 51.32 | 14.59 | 51.50 | 3.53 | TRA1 |
| GTTTAAGTTAAATAAAA | 14.68 | 7.16 | 32.29 | 43.99 | 10.92 | 38.14 | 3.49 | IGF1R |
| GATATGTAAAAACTGTC | 110.07 | 250.62 | 678.17 | 579.24 | 180.34 | 628.71 | 3.49 | CLCA4 |
| CAACCATCATCTTCCAC | 14.68 | 7.16 | 38.75 | 36.66 | 10.92 | 37.71 | 3.45 |  |
| ACAAAGTTGTGCAAAAC | 22.01 | 21.48 | 96.88 | 51.32 | 21.75 | 74.10 | 3.41 | CBR3 |
| GTAAAGACTGCTTTATT | 14.68 | 14.32 | 32.29 | 65.99 | 14.50 | 49.14 | 3.39 | COX7A2 |
| AATTACTTCCTCACTTT | 7.34 | 7.16 | 19.38 | 29.33 | 7.25 | 24.35 | 3.36 | CHMP1.5 |
| GATTTTTACTGCAAAAA | 7.34 | 7.16 | 19.38 | 29.33 | 7.25 | 24.35 | 3.36 | LASS3 |
| GGCTTAGGATGTGAATG | 7.34 | 7.16 | 19.38 | 29.33 | 7.25 | 24.35 | 3.36 | RASIP1 |
| GGGATAAAATAGCTGTT | 7.34 | 7.16 | 19.38 | 29.33 | 7.25 | 24.35 | 3.36 | TCF20 |
| GTTTAATTAAAGGCAAA | 7.34 | 7.16 | 19.38 | 29.33 | 7.25 | 24.35 | 3.36 |  |
| TCATACAGTTTGTGAAA | 7.34 | 7.16 | 19.38 | 29.33 | 7.25 | 24.35 | 3.36 | RPP38 |
| ATTTTTATTCTCTTGCT | 7.34 | 21.48 | 45.21 | 51.32 | 14.41 | 48.27 | 3.35 | HNRPA3 |
| AATAAACTTTGCTGTGG | 22.01 | 7.16 | 45.21 | 51.32 | 14.59 | 48.27 | 3.31 | LOC129607 |
| ATTATCACATTCTGCCA | 7.34 | 7.16 | 25.84 | 22.00 | 7.25 | 23.92 | 3.30 | CSNK1A1 |
| TAAATGTCCTGTTGTAA | 7.34 | 7.16 | 25.84 | 22.00 | 7.25 | 23.92 | 3.30 |  |
| TACATTTTCATATTAAA | 7.34 | 7.16 | 25.84 | 22.00 | 7.25 | 23.92 | 3.30 |  |
| TGGAAATGACCCAAACA | 7.34 | 7.16 | 25.84 | 22.00 | 7.25 | 23.92 | 3.30 | COL1A1 |
| ATTTTTAAATAACCTGT | 14.68 | 14.32 | 51.67 | 43.99 | 14.50 | 47.83 | 3.30 | SF3B1 |
| ATTAAAAAAAAAAAAAA | 22.01 | 14.32 | 64.59 | 51.32 | 18.17 | 57.96 | 3.19 | PBX3 |
| ACTCTAGACACTTGGTG | 7.34 | 14.32 | 32.29 | 36.66 | 10.83 | 34.48 | 3.18 | ENO1 |
| TCATCATCAGACTCCTC | 14.68 | 7.16 | 32.29 | 36.66 | 10.92 | 34.48 | 3.16 |  |
| AAGCAAGAATGACCAAT | 7.34 | 14.32 | 38.75 | 29.33 | 10.83 | 34.04 | 3.14 | ARRDC3 |
| ACTTTTCCAAAAAAAAA | 7.34 | 14.32 | 38.75 | 29.33 | 10.83 | 34.04 | 3.14 |  |
| GTGATTTTTAGTTTTTT | 7.34 | 14.32 | 38.75 | 29.33 | 10.83 | 34.04 | 3.14 | ARHGAP5 |
| TAACTGCCTCAAGGACC | 7.34 | 14.32 | 38.75 | 29.33 | 10.83 | 34.04 | 3.14 | HSD17B8 |
| GTGGATGGACTGAAGTG | 14.68 | 14.32 | 38.75 | 51.32 | 14.50 | 45.04 | 3.11 | GPR175 |
| CATTACTAACTTTATCA | 7.34 | 21.48 | 45.21 | 43.99 | 14.41 | 44.60 | 3.10 |  |
| AACTGTATCAAAATTCT | 14.68 | 14.32 | 51.67 | 36.66 | 14.50 | 44.17 | 3.05 | SLC30A7 |
| GTTTTCCATAGTTGCCT | 22.01 | 28.64 | 58.13 | 95.32 | 25.33 | 76.72 | 3.03 | ATP10B |
| ACTGGTACGTGTAAAAA | 22.01 | 14.32 | 58.13 | 51.32 | 18.17 | 54.73 | 3.01 |  |
| AGGCATTTTATTTGTAA | 66.04 | 42.96 | 187.30 | 139.31 | 54.50 | 163.31 | 3.00 | RPS15A |
| TGATGATCATTTGTGAA | 14.68 | 28.64 | 71.05 | 58.66 | 21.66 | 64.85 | 2.99 | SEC3L1 |
| GGGCCCCAAAGCACTGC | 22.01 | 28.64 | 90.42 | 58.66 | 25.33 | 74.54 | 2.94 | MGC13170 |
| TACATCCGAATGCTAAA | 66.04 | 35.80 | 155.01 | 139.31 | 50.92 | 147.16 | 2.89 | MTPN |
| CAATAAATGTTTTGGTT | 58.70 | 42.96 | 167.93 | 124.65 | 50.83 | 146.29 | 2.88 |  |
| GCTAAGGAGATTGGTGC | 168.78 | 179.01 | 581.29 | 417.93 | 173.89 | 499.61 | 2.87 | RAC1 |
| AAAACTTTGTCTAATTT | 7.34 | 7.16 | 19.38 | 22.00 | 7.25 | 20.69 | 2.85 | PPP3CA |
| AGCCAGTCCGTTTGGGA | 7.34 | 7.16 | 19.38 | 22.00 | 7.25 | 20.69 | 2.85 | GM2A |
| ATAAAAAGTTATTTCCT | 7.34 | 7.16 | 19.38 | 22.00 | 7.25 | 20.69 | 2.85 |  |
| CTAATAAAATTAAGATT | 7.34 | 7.16 | 19.38 | 22.00 | 7.25 | 20.69 | 2.85 | BLP1 |
| CTTTTAAAAAATGCTTT | 7.34 | 7.16 | 19.38 | 22.00 | 7.25 | 20.69 | 2.85 | TPD52 |
| GACTTAAGGTTTCATCC | 7.34 | 7.16 | 19.38 | 22.00 | 7.25 | 20.69 | 2.85 | GTF2I |
| GAGAAATGGGCTGGCCC | 7.34 | 7.16 | 19.38 | 22.00 | 7.25 | 20.69 | 2.85 | POLE |
| GGTGAAGACAAAAAAAA | 7.34 | 7.16 | 19.38 | 22.00 | 7.25 | 20.69 | 2.85 |  |
| TATCCATACCTGGATTT | 7.34 | 7.16 | 19.38 | 22.00 | 7.25 | 20.69 | 2.85 | GCSH |
| TGATAAAGAGGGACCTT | 7.34 | 7.16 | 19.38 | 22.00 | 7.25 | 20.69 | 2.85 | SRP72 |
| TGCAATTCCTTATAGAC | 7.34 | 7.16 | 19.38 | 22.00 | 7.25 | 20.69 | 2.85 | RPL39 |
| TGCTACGAAAAAAAAAA | 7.34 | 7.16 | 19.38 | 22.00 | 7.25 | 20.69 | 2.85 |  |
| TGTCACGAGTTTTTGTT | 7.34 | 7.16 | 19.38 | 22.00 | 7.25 | 20.69 | 2.85 | FLJ10539 |
| TTTATGGGTTATTAAAA | 7.34 | 7.16 | 19.38 | 22.00 | 7.25 | 20.69 | 2.85 | CPM |
| GTGAAACTCCGTCTCTA | 7.34 | 14.32 | 32.29 | 29.33 | 10.83 | 30.81 | 2.85 | ARL10A |
| TATATTTACACATCTGA | 7.34 | 14.32 | 32.29 | 29.33 | 10.83 | 30.81 | 2.85 | LGR4 |
| CTCTGAGGTACTACTGC | 14.68 | 21.48 | 58.13 | 43.99 | 18.08 | 51.06 | 2.82 | PAK1 |
| GAGAATTAATCCCACCT | 22.01 | 28.64 | 83.96 | 58.66 | 25.33 | 71.31 | 2.82 | M11S1 |
| TTTATTTAGCAGACAAG | 161.44 | 143.21 | 355.23 | 483.92 | 152.32 | 419.58 | 2.75 | LCN2 |
| TTAGATCGTTGAAACCC | 51.37 | 50.12 | 116.26 | 161.31 | 50.74 | 138.78 | 2.73 | TM4SF6 |
| AGCAGGCTCAGCCTAGG | 36.69 | 28.64 | 96.88 | 80.65 | 32.67 | 88.77 | 2.72 | S100P |
| TGAAATGAAGGGTGCCT | 22.01 | 21.48 | 58.13 | 58.66 | 21.75 | 58.39 | 2.69 | LOC134492 |
| GTCTTTCACCCAGCCAG | 14.68 | 21.48 | 45.21 | 51.32 | 18.08 | 48.27 | 2.67 | ZDHHC13 |
| CAAGTGGCAAACTCCGG | 14.68 | 21.48 | 51.67 | 43.99 | 18.08 | 47.83 | 2.65 | KIAA1049 |
| GTAAGTGTACTGGAAAG | 410.93 | 343.70 | 981.73 | 1011.83 | 377.32 | 996.78 | 2.64 |  |
| GGTGTATATGGAGCCCT | 14.68 | 14.32 | 38.75 | 36.66 | 14.50 | 37.71 | 2.60 | STOML2 |
| GAATCCAACTGCTTCGA | 29.35 | 35.80 | 83.96 | 80.65 | 32.58 | 82.31 | 2.53 | NDUFB11 |
| AGAATAAAATACTGGCG | 80.72 | 50.12 | 161.47 | 168.64 | 65.42 | 165.05 | 2.52 | CYB5-M |
| AGCAGAACCGCCGAGGG | 66.04 | 57.28 | 142.09 | 168.64 | 61.66 | 155.37 | 2.52 | BJ-TSA-9 |
| TCAATCAAGATGGGTGA | 36.69 | 42.96 | 96.88 | 102.65 | 39.83 | 99.77 | 2.51 | YWHAH |
| CCTGTAATCCCAGATAC | 14.68 | 14.32 | 32.29 | 36.66 | 14.50 | 34.48 | 2.38 | LOC55974 |
| CTAAATATTCTTTCCTA | 14.68 | 14.32 | 32.29 | 36.66 | 14.50 | 34.48 | 2.38 | CSPG6 |
| GAATGGGGCTAAGGTGG | 14.68 | 14.32 | 32.29 | 36.66 | 14.50 | 34.48 | 2.38 | LOC93109 |
| GTTGTGGTTAATCTGCT | 36.69 | 50.12 | 103.34 | 102.65 | 43.41 | 103.00 | 2.37 |  |
| ATTGATCTTGATGGATT | 58.70 | 57.28 | 135.63 | 131.98 | 57.99 | 133.81 | 2.31 | RPA3 |
| ACCTTGTGCCCGATTCT | 95.39 | 107.41 | 238.97 | 219.96 | 101.40 | 229.47 | 2.26 | SORD |
| GGTCACTGAGGCTTTTT | 51.37 | 50.12 | 109.80 | 109.98 | 50.74 | 109.89 | 2.17 |  |
