# Supplementary material for: Comprehensive serial analysis of gene expression of the cervical transcriptome
Source: BMC Genomics. 2007 Jun 1;8:142. doi: 10.1186/1471-2164-8-142 (PMC1899502; doi:10.1186/1471-2164-8-142)
Supplement: Additional File 2 — Supplemental Table 2. Decreased expression in CIN III (≥15 tpm and ≥2 fold change) [file 1471-2164-8-142-S2.doc]

**Supplemental Table 2: Decreased expression in CINIII (>15tpm and >2 fold change)**

| **Tag** | **N1** | **N2** | **C1** | **C2** | **Ave N** | **Ave CINIII** | **Fold Change** | **Symbol** |
| --- | --- | --- | --- | --- | --- | --- | --- | --- |
| TTTTCTGAAAACATAAC | 102.73 | 57.28 | 0.00 | 0.00 | 80.01 | 0.00 | 80.01 | TXN |
| CCCTTCCCCGGCGGAGG | 22.01 | 128.89 | 0.00 | 0.00 | 75.45 | 0.00 | 75.45 | MINK1 |
| TAGGATGGGGTTCTTGT | 29.35 | 42.96 | 0.00 | 0.00 | 36.16 | 0.00 | 36.16 | ATP1B3 |
| TAGGAGAATCCAAGCGA | 29.35 | 28.64 | 0.00 | 0.00 | 29.00 | 0.00 | 29.00 | VDR |
| ACCGTATTCCATCCCAG | 22.01 | 35.80 | 0.00 | 0.00 | 28.91 | 0.00 | 28.91 |  |
| GCCCTGCAGGCAAGCAA | 22.01 | 35.80 | 0.00 | 0.00 | 28.91 | 0.00 | 28.91 | FGFR3 |
| GGTAGCTCAGGGGAGGA | 22.01 | 35.80 | 0.00 | 0.00 | 28.91 | 0.00 | 28.91 | SPINL |
| GGTCACACTATGATGCA | 29.35 | 21.48 | 0.00 | 0.00 | 25.42 | 0.00 | 25.42 | UQCRFS1 |
| ACCCGAGAGGTGCAGAC | 22.01 | 28.64 | 0.00 | 0.00 | 25.33 | 0.00 | 25.33 | RPS3A |
| TTTTCTCAGTTTGGAGG | 22.01 | 28.64 | 0.00 | 0.00 | 25.33 | 0.00 | 25.33 | NID67 |
| AATCCAAGAGGCAGAGC | 146.76 | 21.48 | 0.00 | 7.33 | 84.12 | 3.67 | 22.95 | DDOST |
| AAATTACTGTAAAGAAC | 22.01 | 21.48 | 0.00 | 0.00 | 21.75 | 0.00 | 21.75 | RBM22 |
| AACTAGAAAACCACTTA | 22.01 | 21.48 | 0.00 | 0.00 | 21.75 | 0.00 | 21.75 | TIMM23 |
| AGAGGGATGAGGCAACC | 22.01 | 21.48 | 0.00 | 0.00 | 21.75 | 0.00 | 21.75 | GJB2 |
| CCTGTAATCCCACCACT | 22.01 | 21.48 | 0.00 | 0.00 | 21.75 | 0.00 | 21.75 | EIF3S2 |
| GAAATGGTGTTTATTTA | 22.01 | 21.48 | 0.00 | 0.00 | 21.75 | 0.00 | 21.75 |  |
| GGCAAAAAAAAAAAAAA | 22.01 | 21.48 | 0.00 | 0.00 | 21.75 | 0.00 | 21.75 | MOV10L1 |
| GTCCCTGCCTTAGGCCT | 80.72 | 136.05 | 12.92 | 0.00 | 108.38 | 6.46 | 16.78 | GSTM1 |
| TGGAACTGTGAGTCGAA | 29.35 | 64.44 | 6.46 | 0.00 | 46.90 | 3.23 | 14.52 | CGI-30 |
| GCGAGTCTCCGTGCTGG | 51.37 | 50.12 | 0.00 | 7.33 | 50.74 | 3.67 | 13.84 | FEM1A |
| TTTTGTTGCTCAGTGTT | 58.70 | 21.48 | 6.46 | 0.00 | 40.09 | 3.23 | 12.41 | PTGER4 |
| TCTATTGATGTGTATGC | 44.03 | 28.64 | 6.46 | 0.00 | 36.34 | 3.23 | 11.25 | LXN |
| GGTAAAATTATTAAATA | 36.69 | 35.80 | 6.46 | 0.00 | 36.25 | 3.23 | 11.22 | TSFM |
| TCCATAAGGAGGAAGGG | 29.35 | 42.96 | 6.46 | 0.00 | 36.16 | 3.23 | 11.20 | MCFD2 |
| CCTGGAGCGCCTTCTGC | 44.03 | 35.80 | 0.00 | 7.33 | 39.92 | 3.67 | 10.89 |  |
| GTTTTCATTCAAGGAAA | 44.03 | 21.48 | 6.46 | 0.00 | 32.75 | 3.23 | 10.14 |  |
| GGGCAGGGGCCCAGTTC | 22.01 | 42.96 | 6.46 | 0.00 | 32.49 | 3.23 | 10.06 | PCBP4 |
| GACTCGCAGACACCGGG | 22.01 | 50.12 | 0.00 | 7.33 | 36.07 | 3.67 | 9.84 | SPEN |
| AAATGAGGGACAAAGAA | 36.69 | 21.48 | 6.46 | 0.00 | 29.09 | 3.23 | 9.01 | NPC1 |
| GCATCTGTTTACATTTA | 36.69 | 21.48 | 6.46 | 0.00 | 29.09 | 3.23 | 9.01 | ELOVL5 |
| GGGCAGATGCCACAAGC | 36.69 | 21.48 | 6.46 | 0.00 | 29.09 | 3.23 | 9.01 | FLJ21919 |
| TCATTCATTAAATGTTG | 36.69 | 21.48 | 6.46 | 0.00 | 29.09 | 3.23 | 9.01 | GIMAP6 |
| TTTAATAAACGGAGATT | 36.69 | 21.48 | 6.46 | 0.00 | 29.09 | 3.23 | 9.01 | SYNGAP1 |
| AAAATAAACTTGAATAA | 29.35 | 28.64 | 6.46 | 0.00 | 29.00 | 3.23 | 8.98 | PTEN |
| CACTGATGGACAGAAGA | 29.35 | 35.80 | 0.00 | 7.33 | 32.58 | 3.67 | 8.89 | GRB2 |
| AGTCCTGCTTCTAGCTC | 22.01 | 42.96 | 0.00 | 7.33 | 32.49 | 3.67 | 8.86 | CDKN2B |
| TGGGAACATAAGTTATG | 51.37 | 57.28 | 12.92 | 0.00 | 54.32 | 6.46 | 8.41 | ELL2 |
| GAAAGCATACCTCAGTG | 36.69 | 21.48 | 0.00 | 7.33 | 29.09 | 3.67 | 7.93 | AP4B1 |
| TAATAATACAAGAACTG | 36.69 | 21.48 | 0.00 | 7.33 | 29.09 | 3.67 | 7.93 | KIAA0431 |
| TCTCTGGTTTCAGAATT | 36.69 | 21.48 | 0.00 | 7.33 | 29.09 | 3.67 | 7.93 | DZIP3 |
| GTACTAGTGTTTTTTAG | 80.72 | 28.64 | 6.46 | 7.33 | 54.68 | 6.90 | 7.93 | CCL2 |
| CACACCCATTAAAAATA | 29.35 | 28.64 | 0.00 | 7.33 | 29.00 | 3.67 | 7.91 | MGC15875 |
| TGTATTTGTAATAGATT | 29.35 | 28.64 | 0.00 | 7.33 | 29.00 | 3.67 | 7.91 | ALS2CR4 |
| TTCACTTATGGCTGGTT | 29.35 | 28.64 | 0.00 | 7.33 | 29.00 | 3.67 | 7.91 | PAPLN |
| ACACACAGCAACATCCC | 22.01 | 35.80 | 0.00 | 7.33 | 28.91 | 3.67 | 7.89 | C20orf42 |
| TTTATGTCATAGACGTA | 29.35 | 21.48 | 6.46 | 0.00 | 25.42 | 3.23 | 7.87 | QDPR |
| AGCCACCGTGCCCAGCC | 22.01 | 28.64 | 6.46 | 0.00 | 25.33 | 3.23 | 7.84 | SGEF |
| AGTTAGAGAGCTGGTGA | 22.01 | 28.64 | 6.46 | 0.00 | 25.33 | 3.23 | 7.84 | LEPROTL1 |
| ATGGCAACAGAAACCAA | 22.01 | 28.64 | 6.46 | 0.00 | 25.33 | 3.23 | 7.84 | AQP1 |
| GCAATTTAACCACATTT | 22.01 | 28.64 | 6.46 | 0.00 | 25.33 | 3.23 | 7.84 | SLCO2A1 |
| GCTTTTCCTGTGTATAT | 22.01 | 78.76 | 6.46 | 7.33 | 50.39 | 6.90 | 7.31 | FLJ13910 |
| AGATTATATGTGGTAAA | 51.37 | 42.96 | 12.92 | 0.00 | 47.16 | 6.46 | 7.30 | KIAA0114 |
| AGCTTTTCTCGCAGGAA | 29.35 | 21.48 | 0.00 | 7.33 | 25.42 | 3.67 | 6.93 | DUSP3 |
| GCACCATAATACATTTT | 29.35 | 21.48 | 0.00 | 7.33 | 25.42 | 3.67 | 6.93 |  |
| CTGCTGAAGTCCTTCTC | 22.01 | 28.64 | 0.00 | 7.33 | 25.33 | 3.67 | 6.91 |  |
| GGTTCCTGGTGCCCCTT | 22.01 | 28.64 | 0.00 | 7.33 | 25.33 | 3.67 | 6.91 | FLJ13725 |
| TGGAGCACCAGCCTCGG | 22.01 | 28.64 | 0.00 | 7.33 | 25.33 | 3.67 | 6.91 | OSBPL3 |
| AATAAATGCCCAGTAGA | 22.01 | 21.48 | 6.46 | 0.00 | 21.75 | 3.23 | 6.73 | RHD |
| AGGTATTGTGAAACAGC | 22.01 | 21.48 | 6.46 | 0.00 | 21.75 | 3.23 | 6.73 | CCND1 |
| CCTATAATCCTAGCACT | 22.01 | 21.48 | 6.46 | 0.00 | 21.75 | 3.23 | 6.73 | KIAA0934 |
| CTGAAGTCTATTACAAG | 22.01 | 21.48 | 6.46 | 0.00 | 21.75 | 3.23 | 6.73 | CXorf26 |
| TGAACTTTCCTGGGCAC | 22.01 | 21.48 | 6.46 | 0.00 | 21.75 | 3.23 | 6.73 | SLC27A1 |
| TGAGTTGGGTCAAGTGC | 22.01 | 21.48 | 6.46 | 0.00 | 21.75 | 3.23 | 6.73 | SURF6 |
| TGATTCACTTCCACTCC | 22.01 | 21.48 | 6.46 | 0.00 | 21.75 | 3.23 | 6.73 |  |
| TGGTTGGTTTATGTGAC | 22.01 | 21.48 | 6.46 | 0.00 | 21.75 | 3.23 | 6.73 | ARHGAP26 |
| TTCCTGCTCTTGTCCTG | 22.01 | 21.48 | 6.46 | 0.00 | 21.75 | 3.23 | 6.73 |  |
| TAGCTAATATTTTTTGG | 36.69 | 50.12 | 12.92 | 0.00 | 43.41 | 6.46 | 6.72 | SFRS7 |
| GAACCTGCTGGCATTGG | 58.70 | 71.60 | 12.92 | 7.33 | 65.15 | 10.12 | 6.44 |  |
| AATGTATTGTACACTGA | 29.35 | 64.44 | 0.00 | 14.66 | 46.90 | 7.33 | 6.40 | TNS |
| TGAGCGTGGGAGTGAGG | 51.37 | 35.80 | 6.46 | 7.33 | 43.58 | 6.90 | 6.32 | FUK |
| ACGCTGAATAAATGTCT | 36.69 | 136.05 | 12.92 | 14.66 | 86.37 | 13.79 | 6.26 |  |
| AAGGCAGAAGGTTTAGC | 22.01 | 21.48 | 0.00 | 7.33 | 21.75 | 3.67 | 5.93 | SYVN1 |
| CTACCTGACCTTAGTTT | 22.01 | 21.48 | 0.00 | 7.33 | 21.75 | 3.67 | 5.93 | MCC |
| CTAGCCCCACGAAACTG | 22.01 | 21.48 | 0.00 | 7.33 | 21.75 | 3.67 | 5.93 |  |
| GAAAAATTAATTATATG | 22.01 | 21.48 | 0.00 | 7.33 | 21.75 | 3.67 | 5.93 | ANKRD17 |
| TCTCAGATGAGGAGGGA | 22.01 | 21.48 | 0.00 | 7.33 | 21.75 | 3.67 | 5.93 | CYP27A1 |
| ACCAGCTGTCCAGGGGC | 51.37 | 71.60 | 6.46 | 14.66 | 61.49 | 10.56 | 5.82 | MBD6 |
| GACTTGGCCTCTGGAGT | 58.70 | 21.48 | 6.46 | 7.33 | 40.09 | 6.90 | 5.81 | AMICA1 |
| GTTGGGAAGACGTCACC | 44.03 | 28.64 | 12.92 | 0.00 | 36.34 | 6.46 | 5.63 | STEAP |
| CCACTGCACTCCAGACT | 44.03 | 28.64 | 6.46 | 7.33 | 36.34 | 6.90 | 5.27 | Shax3 |
| GCCATCTTTATTCCCAG | 29.35 | 35.80 | 12.92 | 0.00 | 32.58 | 6.46 | 5.04 |  |
| GCGAAAAAAAAAAAAAA | 29.35 | 35.80 | 12.92 | 0.00 | 32.58 | 6.46 | 5.04 | PDXK |
| GGAAAAAAAAAATCCTC | 29.35 | 35.80 | 12.92 | 0.00 | 32.58 | 6.46 | 5.04 |  |
| GCTTGATGTGAAGACAG | 51.37 | 50.12 | 12.92 | 7.33 | 50.74 | 10.12 | 5.01 | PAIP2 |
| CCAGGCTGCGTCCCTCC | 36.69 | 64.44 | 12.92 | 7.33 | 50.57 | 10.12 | 4.99 | ITGB5 |
| CCCAAACTTTGAGAATT | 36.69 | 28.64 | 6.46 | 7.33 | 32.67 | 6.90 | 4.74 | KCTD12 |
| TTCACCAGGGAACAGGA | 36.69 | 28.64 | 6.46 | 7.33 | 32.67 | 6.90 | 4.74 | ACTR1A |
| TTTTCCACTTTAAAAAA | 36.69 | 28.64 | 6.46 | 7.33 | 32.67 | 6.90 | 4.74 | RNF13 |
| GATTGCGGATATTTACC | 29.35 | 35.80 | 6.46 | 7.33 | 32.58 | 6.90 | 4.72 | LOC388962 |
| AAAATTTATTAGATACC | 22.01 | 42.96 | 6.46 | 7.33 | 32.49 | 6.90 | 4.71 | GNG10 |
| AAATATGTGTTGTTTTC | 22.01 | 42.96 | 6.46 | 7.33 | 32.49 | 6.90 | 4.71 | ESAM |
| GAAGGGACGGATATTTT | 29.35 | 28.64 | 12.92 | 0.00 | 29.00 | 6.46 | 4.49 |  |
| TGGTTTCACTTCCACTC | 29.35 | 28.64 | 12.92 | 0.00 | 29.00 | 6.46 | 4.49 |  |
| GTGAGAGCAGGATTGAG | 36.69 | 50.12 | 12.92 | 7.33 | 43.41 | 10.12 | 4.29 |  |
| GATACTGAGGCATAGCT | 36.69 | 21.48 | 6.46 | 7.33 | 29.09 | 6.90 | 4.22 | C19orf12 |
| TAACTTTGCCATCAGTT | 29.35 | 28.64 | 6.46 | 7.33 | 29.00 | 6.90 | 4.21 |  |
| ATTTTGTAACCCAGATT | 22.01 | 35.80 | 6.46 | 7.33 | 28.91 | 6.90 | 4.19 | RBL2 |
| GGGGCCCCCTCACCTTG | 22.01 | 35.80 | 6.46 | 7.33 | 28.91 | 6.90 | 4.19 | SSNA1 |
| TCTGTTGTTCAAGTAAA | 22.01 | 35.80 | 6.46 | 7.33 | 28.91 | 6.90 | 4.19 | LOC284454 |
| GATATGTTATACTGAAC | 29.35 | 57.28 | 6.46 | 14.66 | 43.32 | 10.56 | 4.10 | COL17A1 |
| TGAGATTGATATGGATT | 212.80 | 128.89 | 32.29 | 51.32 | 170.85 | 41.81 | 4.09 | HPGD |
| CATAGAGCCACGGGAGG | 80.72 | 85.93 | 12.92 | 29.33 | 83.32 | 21.12 | 3.94 | C15orf12 |
| GGGAGTAATAGGACCAG | 36.69 | 71.60 | 12.92 | 14.66 | 54.15 | 13.79 | 3.93 | PTPRA |
| CCCTCTCTGTACATCTT | 51.37 | 78.76 | 19.38 | 14.66 | 65.07 | 17.02 | 3.82 | FLJ22582 |
| GGACCAGGCTGGCCTGA | 51.37 | 78.76 | 19.38 | 14.66 | 65.07 | 17.02 | 3.82 | FLJ20186 |
| GTGTTAGCGCAAAAGTT | 44.03 | 85.93 | 19.38 | 14.66 | 64.98 | 17.02 | 3.82 |  |
| GATTTTTGTGGTGTGGG | 51.37 | 50.12 | 19.38 | 7.33 | 50.74 | 13.35 | 3.80 | GJA1 |
| CGGGAGACATCTTTGGC | 29.35 | 21.48 | 6.46 | 7.33 | 25.42 | 6.90 | 3.69 |  |
| CTCTGTAGTGAGAGGAG | 29.35 | 21.48 | 6.46 | 7.33 | 25.42 | 6.90 | 3.69 | SMAD2 |
| GGTTCAAGGCCCTGGCC | 29.35 | 21.48 | 6.46 | 7.33 | 25.42 | 6.90 | 3.69 | FLJ22635 |
| GTAGACTCACAGCTGCC | 29.35 | 21.48 | 6.46 | 7.33 | 25.42 | 6.90 | 3.69 | BAT2 |
| TATGTTAATGTATGAAA | 29.35 | 21.48 | 6.46 | 7.33 | 25.42 | 6.90 | 3.69 | DUSP16 |
| TTTATACACCTATCCCC | 29.35 | 21.48 | 6.46 | 7.33 | 25.42 | 6.90 | 3.69 |  |
| TTTTGCCTAATATATTC | 29.35 | 21.48 | 6.46 | 7.33 | 25.42 | 6.90 | 3.69 | AKAP9 |
| CCCGGCTAATTTTTTTT | 22.01 | 28.64 | 6.46 | 7.33 | 25.33 | 6.90 | 3.67 | CUL1 |
| TCCAGGGCCGCCGGGTC | 22.01 | 28.64 | 6.46 | 7.33 | 25.33 | 6.90 | 3.67 | ASPSCR1 |
| TAAAGATCCTCTGTAAA | 51.37 | 100.25 | 19.38 | 22.00 | 75.81 | 20.69 | 3.66 | PDCD4 |
| TTCCCTGGGAAGACGGG | 29.35 | 71.60 | 12.92 | 14.66 | 50.48 | 13.79 | 3.66 | ZBTB4 |
| ACAATGGTATAACAAGG | 44.03 | 28.64 | 12.92 | 7.33 | 36.34 | 10.12 | 3.59 | TMP21 |
| ATGCCCGAGGAGAAGCT | 36.69 | 35.80 | 6.46 | 14.66 | 36.25 | 10.56 | 3.43 | SDOS |
| GTGGCAGAGCGAGACTC | 36.69 | 35.80 | 6.46 | 14.66 | 36.25 | 10.56 | 3.43 | PHCA |
| CCTTGTCCTCTTAAGAC | 95.39 | 71.60 | 19.38 | 29.33 | 83.50 | 24.35 | 3.43 | GM2A |
| GTTGCTGCCCTGGGCTG | 66.04 | 50.12 | 19.38 | 14.66 | 58.08 | 17.02 | 3.41 | NIFIE14 |
| TCAGTGAATTATCTTTT | 88.06 | 50.12 | 19.38 | 22.00 | 69.09 | 20.69 | 3.34 | MRPS14 |
| CCTCTCCTCCCTCTCTG | 66.04 | 71.60 | 19.38 | 22.00 | 68.82 | 20.69 | 3.33 | CENPB |
| CAGGTAAGGTGAACCAG | 22.01 | 21.48 | 6.46 | 7.33 | 21.75 | 6.90 | 3.15 | DNAJA4 |
| CTTGTATGTATATTTCA | 22.01 | 21.48 | 6.46 | 7.33 | 21.75 | 6.90 | 3.15 | THRAP2 |
| CTTTCCCTTGTTACTGT | 22.01 | 21.48 | 6.46 | 7.33 | 21.75 | 6.90 | 3.15 | TLP19 |
| CTTTGGAAATGGAAGGG | 22.01 | 21.48 | 6.46 | 7.33 | 21.75 | 6.90 | 3.15 | C10orf9 |
| GCCTGGTGACCAGCTCC | 22.01 | 21.48 | 6.46 | 7.33 | 21.75 | 6.90 | 3.15 | DAXX |
| GTGGTGGGCACCTGTAG | 22.01 | 21.48 | 6.46 | 7.33 | 21.75 | 6.90 | 3.15 |  |
| TAAAATACTCCACAATA | 22.01 | 21.48 | 6.46 | 7.33 | 21.75 | 6.90 | 3.15 | TOM1L2 |
| TATGTCTACTTTGTAGT | 22.01 | 21.48 | 6.46 | 7.33 | 21.75 | 6.90 | 3.15 | KIAA1240 |
| TCTTTCTTTTTGAAAGA | 22.01 | 21.48 | 6.46 | 7.33 | 21.75 | 6.90 | 3.15 | C4orf8 |
| TGCCTTTAACCAGCTTT | 22.01 | 21.48 | 6.46 | 7.33 | 21.75 | 6.90 | 3.15 | MGC13159 |
| TGGATTTTTGACAAGGA | 22.01 | 21.48 | 6.46 | 7.33 | 21.75 | 6.90 | 3.15 | UBE3B |
| TGGTTAATTTTTTTTTT | 22.01 | 21.48 | 6.46 | 7.33 | 21.75 | 6.90 | 3.15 |  |
| TTACAGCACAATATTTC | 22.01 | 21.48 | 6.46 | 7.33 | 21.75 | 6.90 | 3.15 | LYPLA1 |
| TTGGGTTGTTACAGAGT | 22.01 | 21.48 | 6.46 | 7.33 | 21.75 | 6.90 | 3.15 | TTC4 |
| CAGTACATAAAAAGGGG | 29.35 | 35.80 | 6.46 | 14.66 | 32.58 | 10.56 | 3.08 | IPO11 |
| CTCGTGGGAAAATGTTG | 29.35 | 35.80 | 6.46 | 14.66 | 32.58 | 10.56 | 3.08 | HES2 |
| GGCGGGGCCAGCCACCG | 29.35 | 35.80 | 6.46 | 14.66 | 32.58 | 10.56 | 3.08 | MAST4 |
| GTGGCCACGGCCACCAG | 66.04 | 57.28 | 19.38 | 22.00 | 61.66 | 20.69 | 2.98 |  |
| TTAGCACTGTGGATGGG | 44.03 | 35.80 | 12.92 | 14.66 | 39.92 | 13.79 | 2.89 | RAB35 |
| CTTTTCTTCAAGGAAAG | 36.69 | 42.96 | 12.92 | 14.66 | 39.83 | 13.79 | 2.89 | MDS010 |
| GGAACTTTTAGGGGAAC | 102.73 | 114.57 | 38.75 | 36.66 | 108.65 | 37.71 | 2.88 | SULF2 |
| AAGGTAACTTGGGTTTT | 29.35 | 50.12 | 12.92 | 14.66 | 39.74 | 13.79 | 2.88 | PANK3 |
| ACCCTGGGCACAGGGGA | 29.35 | 28.64 | 12.92 | 7.33 | 29.00 | 10.12 | 2.86 | FLJ13052 |
| GGCCCTAGGCAGACTGC | 29.35 | 28.64 | 12.92 | 7.33 | 29.00 | 10.12 | 2.86 | ZFP36L2 |
| TTGGTCCCCTGCCCTGG | 29.35 | 28.64 | 12.92 | 7.33 | 29.00 | 10.12 | 2.86 |  |
| TGGTTTATTGCGAGTTT | 51.37 | 64.44 | 19.38 | 22.00 | 57.91 | 20.69 | 2.80 | JARID2 |
| TTAAATAAAATATTAAG | 73.38 | 93.09 | 25.84 | 36.66 | 83.23 | 31.25 | 2.66 | HARSL |
| TGACTGGCAGTATTAAG | 95.39 | 121.73 | 38.75 | 43.99 | 108.56 | 41.37 | 2.62 | CD59 |
| CCTGTAATTCCAGCTAC | 29.35 | 42.96 | 12.92 | 14.66 | 36.16 | 13.79 | 2.62 | FLJ10349 |
| CTACTGCACTCCAGCCT | 29.35 | 42.96 | 12.92 | 14.66 | 36.16 | 13.79 | 2.62 | LOC133926 |
| CTTCCGTAGCTCTGACC | 29.35 | 42.96 | 12.92 | 14.66 | 36.16 | 13.79 | 2.62 | DERPC |
| TTGTGAGAATAAATGAG | 80.72 | 114.57 | 38.75 | 36.66 | 97.64 | 37.71 | 2.59 | DLGAP4 |
| AACCCGGGAGGTGGAGC | 73.38 | 78.76 | 32.29 | 29.33 | 76.07 | 30.81 | 2.47 | SLC35E2 |
| GATGTATTCTAAGAGCT | 44.03 | 57.28 | 19.38 | 22.00 | 50.66 | 20.69 | 2.45 | C13orf9 |
| CACTCTGGAATTTGTTG | 29.35 | 35.80 | 12.92 | 14.66 | 32.58 | 13.79 | 2.36 | WDR42A |
| TTCACACACCTATCCCC | 29.35 | 35.80 | 12.92 | 14.66 | 32.58 | 13.79 | 2.36 |  |
