# Supplementary material for: Comprehensive serial analysis of gene expression of the cervical transcriptome
Source: BMC Genomics. 2007 Jun 1;8:142. doi: 10.1186/1471-2164-8-142 (PMC1899502; doi:10.1186/1471-2164-8-142)
Supplement: Additional File 4 — Supplemental Table 3. Cervical Specimen Description based on LEEP cone biopsy Pathology Report [file 1471-2164-8-142-S4.doc]

**Supplemental Table 3: Cervical Specimen Description based on LEEP cone biopsy Pathology Report**

| Normal | N1 | Patient age: 45 |
| --- | --- | --- |
|  |  | LEEP cone biopsy measuring 2.1X2cm excised to a depth of 1cm |
|  |  | Description   - LEEP cone biopsy of the uterine cervix, no residual   dysplasia |
|  | N2 | Patient age: 24 |
|  |  | LEEP cone biopsy measuring 2.1X1.5cm at the base and 0.3cm up the canal |
|  |  | Description  - LEEP cone biopsy negative for dysplasia |
| CIN III | C1 | Patient age: 42 |
|  |  | LEEP cone biopsy of the cervix 1.5cm in diameter and 1cm in depth |
|  |  | Description   - Specimen contains severely dysplastic squamous epithelium (CIN III) - No biopsy margin involvement by dysplastic epithelium is identified |
|  | C2 | Patient age: 23 |
|  |  | LEEP cone biopsy measuring 2.5X2X0.6cm |
|  |  | Description   - severe dysplasia of the squamous epithelium which extend some distance into the endocervical canal and is focally seen close to but clear of the endocervical resection margin - The exocervical resection margin shows mature squamous epithelium - Severe dysplasia of the squamous epithelium (CIN III) - Completely excised in the plane of sectioning |
